# Cellular senescence and regulated cell death in cancer: mechanisms, cross-regulatory networks and therapeutic implications

**DOI:** 10.1186/s13045-026-01847-x

**Published:** 2026-09-29

**Authors:** Linna Du, Yueyue Zhang, Ruohan Liang, Bin Song, Jun Zhou, Xianwu Xia, Chibo Liu, Kongming Wu, Xuan Cao

**Affiliations:** 1 Department of Clinical Laboratory, Taizhou Key Laboratory of Infection and Tumor Immunology, Taizhou Municipal Hospital (Taizhou University Affiliated Municipal Hospital), School of Medicine, Taizhou University, Taizhou, Zhejiang 318000 China; 2 Department of Basic Medicine and Medical Laboratory Science, School of Medicine, Zhejiang Key Laboratory of New Drug Development for Central Nervous System Diseases, Taizhou University, Taizhou, Zhejiang 318000 China; 3 Cancer Center, Shanxi Bethune Hospital, Shanxi Academy of Medical Science, Tongji Shanxi Hospital, Third Hospital of Shanxi Medical University, Taiyuan, Shanxi China; 4 Department of Oncology, Tongji Hospital of Tongji Medical College, Huazhong University of Science and Technology, Wuhan, Hubei China

**Keywords:** Cellular senescence, Regulated cell death, Cancer, SASP, Senolytics, Combination therapy

## Abstract

Cellular senescence and regulated cell death represent two fundamental but intricately connected biological programs that govern tumor cell fate. Cellular senescence can suppress tumor growth by inducing stable cell-cycle arrest and promoting immune clearance, but persistent senescent cells may also drive tumor progression through the senescence-associated secretory phenotype (SASP). Concurrently, multiple modalities of regulated cell death—including apoptosis, ferroptosis, cuproptosis, necroptosis, pyroptosis, parthanatos, and autophagy-dependent cell death—have emerged as critical determinants of therapeutic response. The crosstalk between senescence and cell death is orchestrated by key molecular hubs including p53, mitochondria, autophagy machinery, and epigenetic regulators. In this review, we summarize the hallmarks and classification of cellular senescence in cancer, outline major cell death modalities, discuss the cross-regulatory networks linking senescence and death, and highlight emerging therapeutic strategies that target these interconnected processes for cancer treatment.

**Graphical Abstract:**

**Figure:**
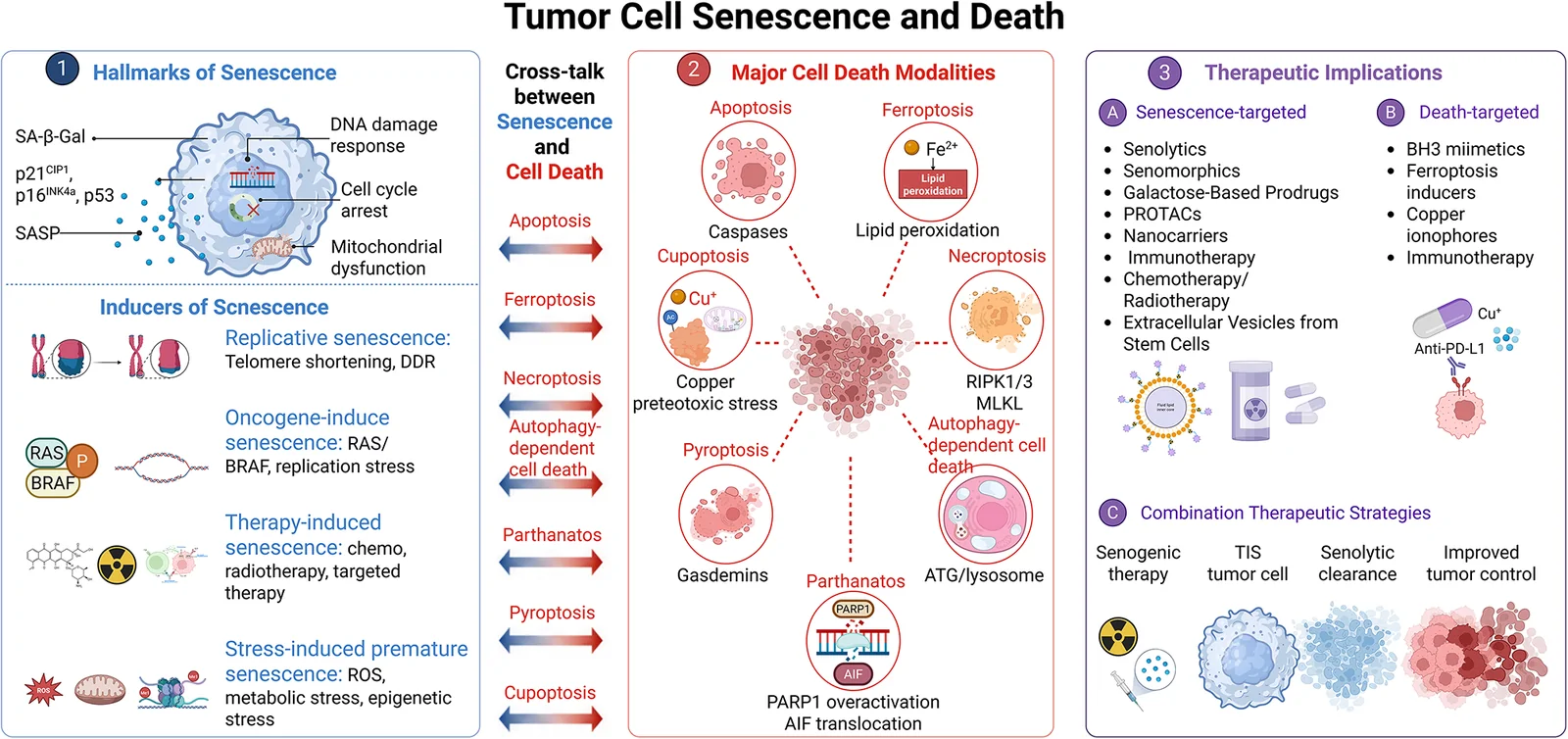

## Introduction

Cancer cells are constantly exposed to different types of stress, including oncogene activation, DNA damage, oxidative stress, nutrient limitation, and anticancer treatment. In response, tumor cells activate a variety of stress-response programs [1, 2]. Adaptive programs—such as antioxidant defenses, DNA repair, chaperone induction, and cytoprotective autophagy—are engaged to restore homeostasis and maintain viability. Cellular senescence and regulated cell death represent fundamentally different outcomes that emerge when adaptation is incomplete: senescence is a durable but viable arrest of severely stressed cells, whereas regulated cell death generally occurs when stress cannot be adequately resolved, or when death signaling overrides the cell’s adaptive capacity [3, 4]. For many years, these two processes were mainly studied separately. Senescence was viewed as a stable growth-arrest program, whereas cell death was regarded as the final elimination of damaged cells. However, recent studies have shown that this view is too simple. In reality, senescence and cell death are closely connected and often influence each other during tumor development and cancer therapy For many years, these two processes were mainly studied separately. Senescence was viewed as a stable growth-arrest program, whereas cell death was regarded as the final elimination of damaged cells. However, recent studies have shown that this view is too simple. In reality, senescence and cell death are closely connected and often influence each other during tumor development and cancer therapy [1, 2].

Cellular senescence is a stable and usually irreversible state of cell-cycle arrest [3, 4]. It can be induced by telomere shortening, oncogenic signaling, DNA damage, oxidative stress, metabolic disturbance, and many anticancer therapies [3–5]. In early tumorigenesis, senescence acts as a protective barrier by preventing the proliferation of damaged or premalignant cells [4]. This tumor-suppressive effect is mainly mediated by the p53/p21 and p16/RB pathways [4, 5]. Senescent cells, however, are not inactive. They remain metabolically active and secrete many cytokines, chemokines, growth factors, and proteases, which together form the senescence-associated secretory phenotype, or SASP [4, 5]. A transient SASP may support immune clearance and tissue repair, but long-term accumulation of senescent cells can remodel the tumor microenvironment, promote inflammation, stimulate invasion and metastasis, and contribute to therapy resistance [5–7]. Therefore, senescence is now widely considered a double-edged process in cancer.

At the same time, tumor cell death is much more complex than classical apoptosis alone [1, 8]. In addition to apoptosis, several other forms of regulated cell death have attracted major attention in cancer research, including ferroptosis, autophagy-dependent cell death, necroptosis, pyroptosis, parthanatos, and the recently described cuproptosis [1, 8, 9]. These death modalities differ in their molecular mechanisms and biological consequences, but all of them may affect tumor progression and therapeutic response [10]. Importantly, the boundary between senescence and death is not fixed. Depending on stress intensity, metabolic state, mitochondrial function, and checkpoint integrity, a tumor cell may enter senescence, remain viable, or switch to a lethal program [1, 2].

This growing understanding has important therapeutic implications. Pro-senescence therapy can suppress tumor growth, but persistent senescent cells may later become harmful if they are not cleared [11, 12]. Likewise, activating non-apoptotic death pathways offers new opportunities for treating apoptosis-resistant tumors [9, 10, 13]. As a result, increasing attention is being paid to therapeutic strategies that combine senescence induction, senolytic clearance, and activation of complementary cell death programs [11, 12, 14].

In this review, we summarize the major features and types of tumor cell senescence, outline the main regulated cell death modalities in cancer, discuss the molecular networks that connect senescence and death, and highlight emerging therapeutic strategies based on these interactions.

Relative to previous reviews on the interplay between cell death and senescence in cancer [3, 4], the present review offers the following distinguishing contributions. First, it is organized around cross-regulatory molecular hubs—p53, mitochondria, autophagy machinery, and epigenetic regulators—rather than as a pathway-by-pathway catalog, providing an integrative fate-decision perspective in which adaptive capacity, stress dose, and the DNA damage response determine the outcome among adaptation, senescence, and death. Second, it systematically covers seven regulated cell death modalities and their respective links to senescence (Sections “Mechanistic links between senescence and apoptosis”–“Mechanistic links between senescence and cuproptosis”), including parthanatos and cuproptosis, which are rarely integrated into senescence-focused discussions. Third, it applies the current Nomenclature Committee on Cell Death conceptual framework rigorously, distinguishing regulated cell death from programmed developmental death, autophagy-dependent cell death from cytoprotective autophagy, and mechanistic links from genuine cell-fate transitions. Fourth, it introduces an explicit three-context framework separating senescence as a premalignant tumor-suppressive barrier, senescence of established malignant cells, and senescence of non-malignant tumor microenvironment cells, and maintains this distinction throughout. Fifth, it provides a critical translational assessment of senescence- and death-targeted strategies, explicitly separating clinical evidence from preclinical findings and identifying specific, testable knowledge gaps (Sections “Combination therapeutic strategies”–“Challenges and limitations of senescence-targeted therapies” and “Conclusion”).

## Cellular senescence in cancer

Cellular senescence in the context of cancer should be distinguished into three biologically discrete situations. First, senescence can act as a tumor-suppressive barrier before or during early tumorigenesis, as exemplified by replicative senescence and oncogene-induced senescence in premalignant cells, such as the durable growth arrest of benign melanocytic nevi harboring BRAF^V600E^ [15]. Second, senescence of established malignant cells, including therapy-induced senescence (TIS) and stress-induced senescence of cancer cells, represents a stress-response fate that can transiently suppress tumor growth but, when persistent, may seed relapse and therapy resistance. Third, senescence of non-malignant cells within the tumor microenvironment—including cancer-associated fibroblasts, endothelial cells, immune cells, mesenchymal stromal cells, and hepatic stellate cells—can indirectly shape tumor growth, immune surveillance, metastasis, and therapeutic response through paracrine signaling and matrix remodeling [16–21]. These three contexts differ in the senescing cell type, the biological timing, and the consequences for tumor development and treatment, and this distinction is maintained explicitly throughout the manuscript.

### Hallmarks of cellular senescence

Cellular senescence is defined foremost by a durable loss of proliferative capacity in cells that remain viable and metabolically active. A core and frequently shared feature is a stable cell-cycle arrest enforced by checkpoint networks centered on the p53/p21 and RB pathways, which prevent damaged, oncogene-activated, or therapy-stressed tumor cells from continuing division [5, 22].

Although persistent DDR signaling is a shared feature of many canonical senescence states—such as replicative senescence triggered by telomere erosion [23, 24], oncogene-induced senescence driven by replication stress [25, 26], and therapy-induced senescence elicited by genotoxic treatment [6, 27]—it is not an obligatory defining criterion. Senescence can also be induced independently of DNA damage [4], through primary triggers including mitochondrial dysfunction, perturbed mitogenic or proteotoxic signaling, inflammatory cytokines, and epigenetic or chromatin remodeling; in these settings, the senescence-associated growth arrest is enforced through p53-, p16INK4a/RB-, or stress-activated pathways without requiring an initiating DDR event [4, 22]. Accordingly, the DDR is presented throughout this review as a common, context-dependent feature that reinforces and shapes the senescence program and its secretory output, rather than as a defining mechanism of all senescent states.

A second hallmark is lysosomal and metabolic remodeling. Senescent tumor cells commonly become enlarged and flattened, show markedly increased lysosomal content and senescence-associated β-galactosidase activity, and undergo extensive metabolic remodeling rather than entering an inactive state [5, 28, 29]; SA-β-gal activity itself was first established as a biomarker of senescent cells in culture and in aging skin in vivo in the landmark study by Dimri et al. [30]. In therapy-induced senescence, this state is also accompanied by hypercatabolic and stress-adaptive metabolic changes that create exploitable vulnerabilities In therapy-induced senescence, this state is also accompanied by hypercatabolic and stress-adaptive metabolic changes that create exploitable vulnerabilities [31].

Another defining feature is persistent DNA-damage signaling coupled to chromatin reorganization. SAHF formation is a hallmark of some—but not all—senescence states: classical work showed that SAHF are linked to RB-dependent silencing of E2F target genes and stabilization of cell-cycle exit [32, 33], but their detection depends on the inducing stimulus and the cell type, being most prominent in oncogene-induced senescence and in human fibroblast models, whereas many murine and human tumor cell senescence programs display more dispersed heterochromatin changes or DNA-methylation alterations without fully formed SAHF. Accordingly, SAHF should be regarded as a context-dependent marker rather than a universal feature of senescence [4, 32]. Beyond SAHF, senescence is also characterized by sustained DNA damage signaling and broad reorganization of chromatin accessibility. In oncogene-driven settings, DNA damage response signaling and heterochromatin remodeling are tightly Beyond SAHF, senescence is also characterized by sustained DNA damage signaling and broad reorganization of chromatin accessibility. In oncogene-driven settings, DNA damage response signaling and heterochromatin remodeling are tightly interconnected, while NOTCH-dependent programs can reshape chromatin structure in both cell-autonomous and non-cell-autonomous manners [26, 34].

A further defining hallmark is SASP. Senescent tumor cells remain highly transcriptionally and biosynthetically active and secrete inflammatory cytokines, chemokines, growth factors, and matrix-remodeling enzymes that profoundly influence the tumor microenvironment [5]. This secretory program means that senescence is not merely a cell-autonomous arrest program, but also a microenvironment-shaping state. In some contexts, senescent tumor cells become highly immunogenic, enhance antigen processing and presentation, and stimulate productive antitumor immunity [35, 36].

Importantly, the immune interface of senescent tumor cells is itself a hallmark with dual consequences. On the one hand, senescence can trigger immune surveillance and facilitate immune-mediated elimination of premalignant or therapy-damaged cells [35, 37]. On the other hand, persistent senescent cells may acquire immune-evasive features, including suppression of NK- and CD8⁺ T-cell responses and remodeling of extracellular signals that permit their survival within tissues and tumors [38, 39].

Finally, tumor cell senescence is marked by phenotypic plasticity. Although it is classically described as irreversible, accumulating evidence indicates that at least a subset of senescent tumor cells can escape growth arrest. This is particularly concerning in cancer because senescence-associated reprogramming can enhance stem-like properties, clonogenic potential, and tumor-initiating capacity after senescence escape [7]. More recent work in breast cancer further supports the view that therapy-induced senescence may act as a transient drug-resistant state, thereby contributing to recurrence [40].

In summary, the hallmarks of cellular senescence extend well beyond growth arrest alone. They comprise stable checkpoint-enforced proliferative arrest, lysosomal and metabolic remodeling, persistent DNA damage and chromatin reorganization, SASP production, immune interaction, and context-dependent plasticity [5, 7, 22, 29, 32, 36]. This integrated view better explains why senescence can function as both a potent tumor-suppressive barrier and, under conditions of persistence or incomplete clearance, a contributor to tumor progression and therapeutic resistance. The core marks are summarized in Table 1.

**Table 1:** Representative hallmarks and markers of tumor cell senescence

Senescence hallmark	Marker	Variation	Function	References
Stable cell-cycle arrest	p16 (CDKN2A); p21 (CDKN1A); activated/acetylated p53; Ki-67	p16↑; p21↑; p53 activity↑; Ki-67↓	Core hallmark of senescence, indicating durable proliferative withdrawal rather than transient quiescence; Ki-67 loss supports a non-cycling state and should be interpreted together with cell-cycle inhibitors.	[30, 31]
Lysosomal expansion / SA-β-Gal activity	SA-β-Gal (GLB1 activity)	↑	Classic and widely used senescence marker reflecting increased lysosomal content and catabolic remodeling; useful in pathology and experimental models, but not fully specific when used alone.	[32]
Persistent DNA damage response / genomic instability signaling	γH2AX; persistent DNA damage foci; ATM/CHK2 signaling	↑	Indicates unresolved or persistent DDR, which stabilizes senescence and contributes to inflammatory signaling and microenvironmental effects.	[33]
Telomere attrition / telomere dysfunction	Telomere-associated DDR foci; dysfunctional DDR+ telomeres	↑	Characteristic of replicative senescence; dysfunctional telomeres trigger p53-dependent growth arrest and act as an intrinsic barrier against malignant progression.	[34]
Epigenetic and chromatin remodeling	SAHF; macroH2A; heterochromatin-associated chromatin compaction	↑ / remodeled	Reflects stable repression of proliferation-promoting genes and transcriptional locking of the senescent state; SAHF is informative but not universal across all senescence programs.	[21]
Nuclear architecture remodeling	Lamin B1 (LMNB1)	↓	Robust nuclear marker of senescence associated with nuclear lamina disruption, chromatin reorganization, and altered nuclear morphology.	[35]
Proteostasis loss / autophagy remodeling	SQSTM1/p62; GATA4; autophagic flux	p62 remodeling; GATA4↑; flux impaired	Impaired selective autophagy promotes accumulation of senescence regulators and SASP-related signaling; proteostasis failure helps maintain senescence and inflammatory output.	[36, 37]
Deregulated nutrient-sensing / metabolic rewiring	mTOR-linked metabolic adaptation; increased autophagy susceptibility	Dysregulated	Reflects altered nutrient sensing and metabolic adaptation in senescent cancer cells, supporting survival, stress tolerance, and context-dependent tumor-promoting effects.	[38]
Mitochondrial dysfunction	ROS; mitochondrial dysfunction; extracellular mtDNA release	↑ / dysfunctional	Mitochondrial remodeling is a major feature of senescence and contributes to redox imbalance, metabolic reprogramming, and immune-modulatory signaling in the tumor microenvironment.	[38, 39]
Altered intercellular communication / SASP	IL-1α / IL-1β; IL-6; IL-8; SASP factors	↑	Defines the secretory phenotype of senescent cells and mediates autocrine and paracrine communication, including reinforcement of senescence and effects on neighboring tumor or stromal cells.	[33, 40]
Chronic inflammation	NF-κB activation; persistent inflammatory cytokine secretion	↑	Sustains the inflammatory arm of senescence and links persistent senescent cells to tumor-promoting microenvironmental remodeling and tissue dysfunction.	[33, 36, 40]
Extracellular matrix remodeling	MMPs; collagen remodeling; matrix density/stiffness changes	↑ / remodeled	Senescent cells can actively remodel the extracellular matrix, thereby promoting invasion, malignant behavior, and altered tumor–stroma interactions.	[41, 42]
Immune / surfaceome remodeling	Senescence-associated surface proteins; PD-L1	↑ / remodeled	Reflects immune-interface changes of senescent tumor cells, including altered surfaceome composition and immune-evasive signaling that can influence immunotherapy response.	[41, 43]

Beyond acting as an upstream trigger of arrest, the DDR can be viewed as a central decision-making hub that integrates diverse stress signals and determines cell fate outcomes. Upon persistent or irreparable DNA damage, ATM/ATR signaling stabilizes p53, which enforces p21-dependent checkpoint arrest and initiates the senescence program. The same DDR–p53 axis can instead drive apoptosis when damage is overwhelming or when p53 dosage, co-factor availability, and mitochondrial priming favor BAX/BAK-mediated mitochondrial outer membrane permeabilization and caspase execution [41, 42]. DDR signaling also intersects with non-apoptotic outcomes, including PARP1 hyperactivation-driven parthanatos (Section “Parthanatos”) and mitotic catastrophe (Section “Mechanistic links between senescence and necroptosis”). Consequently, the final outcome is not dictated by DNA damage per se, but by its dose, duration, and repairability, integrated with p53/p21 levels, mitochondrial status, autophagic activity, and epigenetic context [4, 23, 43, 44]. We therefore regard the DDR as a common yet context-dependent decision hub—explicitly in DDR-proficient settings—rather than a defining mechanism of all senescent states.

### Classification of senescence in cancer: triggering contexts and biological settings

The four categories retained here—replicative senescence, oncogene-induced senescence, therapy-induced senescence, and stress-induced premature senescence—are defined by their triggering context rather than by the identity of the senescing cell, and they should therefore be interpreted within the three-context framework introduced above. Replicative senescence and oncogene-induced senescence primarily operate as tumor-suppressive barriers in premalignant cells before or during early tumorigenesis [15, 25]. Therapy-induced senescence arises following anticancer treatment and can occur in both malignant cells and non-malignant cells of the tumor microenvironment, with opposite consequences for tumor control versus normal-tissue toxicity, which forms the mechanistic basis for the senolytic and senomorphic strategies discussed in Section “Therapeutic strategies: targeting tumor senescence and cell death”. Stress-induced premature senescence is elicited by microenvironmental stress within established tumors. For each category, the senescing cell type, the evidence source, the biological timing, and the expected consequence are summarized in Table 2.

**Table 2:** Classification of senescence in cancer: triggering contexts and biological settings

Category	Typical senescing cell type	Evidence source	Biological timing	Expected consequence
Replicative senescence	Premalignant and normal proliferating cells	Premalignant / non-malignant	Before tumor formation	Tumor-suppressive barrier; bypass requires telomere maintenance
Oncogene-induced senescence	Premalignant cells (e.g., nevi)	Premalignant	Before or during early tumorigenesis	Tumor-suppressive barrier; escape drives malignant progression
Therapy-induced senescence	Malignant cells and non-malignant TME cells	Malignant and non-malignant	Following therapy	Transient tumor control; persistence seeds relapse and toxicity
Stress-induced premature senescence	Malignant cells and stromal cells	Malignant / non-malignant	Within established tumors	Context-dependent; SASP-mediated remodeling and resistance

#### Replicative senescence (RS)

Replicative senescence is initiated by progressive telomere erosion during repeated cell division. When telomeres become critically short or structurally deprotected, chromosome ends are recognized as persistent DNA damage, activating ATM-dependent DNA damage response signaling, stabilizing p53, inducing p21, and ultimately enforcing durable proliferative arrest [23, 24]. In cancer evolution, bypass of this barrier usually requires activation of a telomere maintenance mechanism; genome-scale analyses indicate that telomerase activation is the dominant strategy across human cancers, whereas a smaller subset of tumors relies on alternative lengthening of telomeres (ALT) [45].

#### Oncogene-induced senescence (OIS)

Oncogene-induced senescence (OIS) is a stress response caused by excessively strong mitogenic signaling, especially in the RAS–RAF pathway. Early studies showed that oncogenic RAS can trigger senescence together with increased p53 and p16INK4a levels, suggesting that OIS acts as an intrinsic barrier against tumor development [43]. Benign melanocytic nevi often carry the BRAF Benign melanocytic nevi often carry the BRAF^V600E^ mutation but remain growth-arrested and display senescence markers such as p16 expression and SA-β-Gal activity [15]. Recent studies further indicate that escape from OIS is an actively controlled process. For example, constitutive MOB3A signaling can overcome BRAF- and RAS-induced senescence through activation of the Hippo pathway, thereby promoting the progression from premalignant lesions to malignant tumors [46].

#### Therapy-induced senescence (TIS)

TIS refers to a long-term growth-arrest program elicited by sublethal exposure to anticancer treatments, including DNA-damaging chemotherapy, radiotherapy, and selected targeted agents. A key biological feature of TIS is that senescent tumor cells acquire senescent cell anti-apoptotic pathway (SCAP) dependencies, especially on BCL-2 family proteins, thereby creating a druggable senolytic vulnerability [47].In breast cancer models, CDK4/6 inhibitor-induced senescence displays stronger antigen-presentation and T-cell-recruiting properties than DNA damage-induced senescence, indicating that the immunologic output of TIS is highly therapy-context dependent [48]. However, persistent TIS can also support residual disease. Chemotherapy-induced senescent cells have been shown to contribute to adverse treatment sequelae and cancer relapse [6], and more recent work demonstrated that senescent cancer cells upregulate PD-L1 through an RPN1-dependent glycosylation program, thereby suppressing cytotoxic T-cell activity and facilitating post-treatment immune escape [49]. Consistent with this plasticity, recent breast cancer data indicate that TIS may be reversible and can function as a transient drug-resistant state rather than an absolutely terminal fate [40].

#### Stress-induced premature senescence (SIPS)

Stress-induced premature senescence is induced independently of replicative telomere exhaustion and is particularly relevant to the hostile tumor microenvironment. Oxidative stress alone can trigger ROS-driven DNA damage, activate the p53–p21 pathway, increase SA-β-Gal activity, and suppress clonogenicity in malignant cells, demonstrating that redox imbalance is sufficient to induce a senescence program in cancer stem-like populations [50]. Glucose deprivation activates an AMPK–p53 metabolic checkpoint [51], and disruption of epigenetic regulators such as DNMT3A can drive p16/p21 upregulation and senescence-associated radiosensitization [52]. In addition, microenvironmental stress modifies the senescence phenotype itself; under physiological hypoxia, cells may resist full geroconversion and display restrained SASP output through AMPK-mediated mTOR suppression, supporting the idea that hypoxic, nutrient-restricted tumor cores can generate atypical but biologically relevant SIPS states [53].

### Inducing factors of tumor cellular senescence

**Fig. 1:**
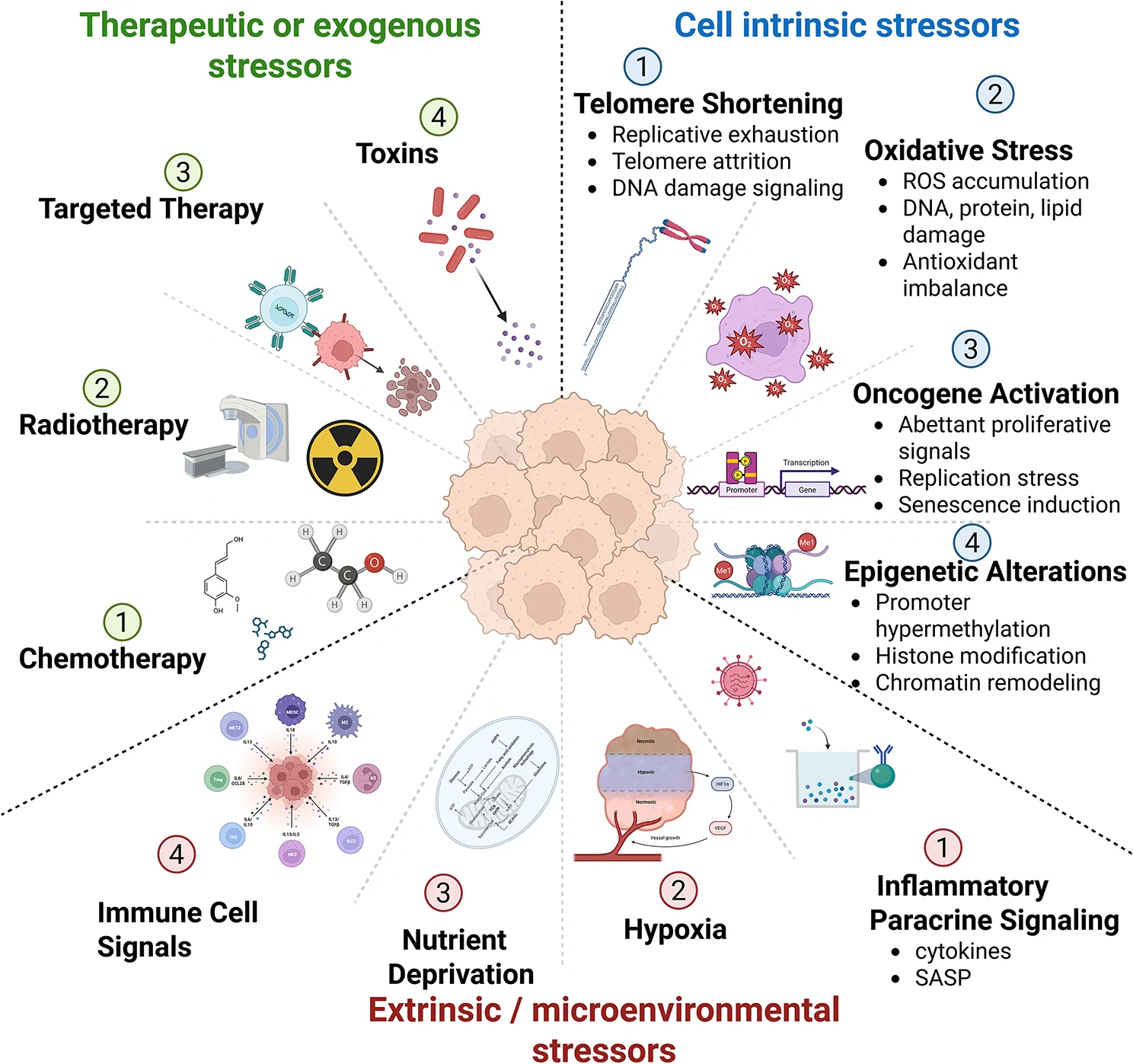
Inducers of cellular senescence in cancer. Cell-intrinsic stressors: telomere dysfunction, oncogene activation, mitochondrial and oxidative stress, metabolic stress, and epigenetic alterations. Extrinsic and microenvironmental stressors: cytokines, paracrine SASP signaling, hypoxia, nutrient deprivation, and immune cell signals. Therapeutic and exogenous stressors: chemotherapy, radiotherapy, targeted therapy, and toxins

The induction of senescence in tumor cells is governed by a constellation of cell-intrinsic stressors, extrinsic and microenvironmental stressors, therapeutic and exogenous stressors, as detailed in Fig. 1. These factors are presented as illustrative triggers of the senescence categories defined in Section “Classification of senescence in cancer: triggering contexts and biological settings”, rather than as a separate classification scheme.

#### Cell-intrinsic stressors

##### Telomere dysfunction

Telomere attrition remains the canonical intrinsic trigger of replicative senescence. Dysfunctional telomeres are recognized as DNA lesions and activate checkpoint signaling [23]. When telomeres become critically short, an ATM–p53–p21 axis enforces durable cell-cycle arrest, thereby functioning as a fundamental barrier to malignant progression [24]. Oxidative damage can engage this checkpoint independently of telomere length: telomeric 8-oxo-guanine is sufficient to drive rapid premature senescence [54], linking oxidative stress directly to the telomere-based senescence machinery.

##### Oncogene activation

Aberrant oncogenic signaling is a core intrinsic trigger of senescence. Activated RAS induces a premature senescence program associated with p53 and p16 accumulation [43]. In vivo, benign melanocytic nevi carrying BRAF^V600E provide strong evidence that oncogenic signaling can produce a durable senescence barrier rather than immediate malignant transformation [15]. Importantly, escape from oncogene-induced senescence is an actively regulated process; constitutive MOB3A signaling bypasses BRAF- and RAS-driven senescence through Hippo-pathway engagement, thereby facilitating malignant progression [46].

##### Mitochondrial and oxidative stress

ROS accumulation causes oxidative DNA damage, including 8-oxo-dG lesions, and promotes lipid peroxidation and mitochondrial dysfunction, all of which can drive senescence-like programs [54]. The eventual cell fate is further shaped by intracellular redox-buffering capacity and antioxidant systems; disruption of redox homeostasis can favor senescence maintenance or, when oxidative injury becomes excessive, promote cell death [55].

##### Metabolic and epigenetic stress

Metabolites imposed from outside the cell can also act intrinsically once imported: sodium butyrate, a short-chain fatty acid that inhibits histone deacetylases, induces SA-β-Gal positivity and p53/p21-dependent senescence and suppresses invasiveness in glioblastoma cells [56]. Such inducers illustrate the overlap between metabolic stress and epigenetic alteration, in that chromatin-level perturbation can reactivate latent senescence programs in otherwise proliferating tumor cells.

#### Extrinsic and microenvironmental stressors

##### Cytokine and immune cell signals

Immune-derived cytokines are bona fide senescence-inducing signals in cancer. IFN-γ and TNF can drive stable growth arrest, increased SA-β-Gal activity, and a distinct SASP program in melanoma cells [57].

##### Paracrine SASP signaling

Although the SASP is often considered a downstream consequence of senescence, it can also propagate senescence through the microenvironment. Conditioned medium from cytokine-induced senescent melanoma cells transmits senescence features to neighboring cells [57]. In an analogous autocrine mode, IL-6 helps maintain the senescent phenotype in pituitary tumor cells and restrains their tumorigenic conversion [58], and in luminal breast cancer cells IL-6 and IL-8 participate in a self-reinforcing inflammatory circuit that sustains senescence-associated features and remodels tumor-cell behavior [59]. Autocrine and paracrine SASP signaling therefore functions both as a maintenance mechanism and as a vehicle for senescence spread within the tumor bed.

##### Hypoxia and nutrient deprivation

Hypoxia, nutrient deprivation, and the metabolic constraints of the tumor microenvironment are increasingly recognized as senescence-relevant cues; however, their direct roles in established tumor-cell senescence models remain less well characterized than that of cytokine signaling.

#### Therapeutic and exogenous stressors

##### Radiotherapy and genotoxic therapy

Ionizing radiation is a classical inducer of tumor-cell senescence because it generates persistent DNA double-strand breaks and long-lasting DDR signaling. In MCF7 breast cancer cells, irradiation induces premature senescence accompanied by SA-β-Gal positivity, cytoskeletal remodeling, lysosomal activation, and sustained cell-cycle arrest [27]. Together with cytotoxic chemotherapy and targeted agents that impose replicative or mitotic stress, radiation exemplifies therapy-induced senescence, in which the redox state of the treated cell influences whether arrested cells mature into senescence or proceed to death [55].

##### Toxins and exogenous carcinogens

Benzo[a]pyrene induces DNA damage, p21 upregulation, SA-β-Gal activity, and nuclear elongation in MCF7 cells [60], while arsenite induces p53/p21-dependent premature senescence in malignant glioblastoma cells [61]. UV-associated photo-oxidative stress represents a further exogenous physical stressor capable of inducing senescence-like programs, although this has been characterized more extensively in primary epithelial systems than in established tumor cell models [62].

##### Withdrawal of exogenous oncogenic drivers

Viral oncogene dependence can unmask a latent senescence program in tumor cells. In cervical carcinoma cells, repression of HPV E6/E7 rapidly reactivates senescence, indicating that viral oncogenes continuously suppress the senescence machinery in established tumors [63]. In this framework, the inducing event is not the accumulation of new damage but the therapeutic withdrawal of an exogenous, virus-derived survival signal—conceptually a targeted-therapy-like intervention that reinstates a senescence program the tumor cell was actively suppressing.

### The consequences of senescence in cancers

Consistent with the three-context framework introduced above, the consequences of senescence are organized below according to whether senescence operates as a tumor-suppressive barrier in premalignant cells, as a stress response of established malignant cells, or in non-malignant cells of the tumor microenvironment. Senescence is a biologically ambivalent process whose net effect on cancer depends on timing, context, and cellular composition [64].

#### Senescence as a tumor suppressor

##### Oncogene-induced senescence (OIS) in tumors

Oncogene-induced senescence (OIS) is widely regarded as an early intrinsic barrier to malignant transformation. Aberrant activation of oncogenic signaling, especially the RAS–RAF axis, provokes replication stress, DNA damage signaling, and stable cell-cycle arrest, thereby restricting the expansion of premalignant clones [25, 43].However, this suppressive state is not irreversible, because active bypass mechanisms such as constitutive MOB3A signaling can overcome BRAF- and RAS-driven senescence and facilitate malignant progression [46]. The canonical example of OIS as a premalignant barrier is the BRAFV600E-driven senescence-like arrest of benign melanocytic nevi [15].

##### Transient SASP promotes immune clearance

A transient and tightly controlled SASP can exert tumor-suppressive effects by recruiting innate and adaptive immune cells to eliminate senescent or damaged cells. Senescent cancer cells can become highly immunogenic, activate dendritic cells, enhance antigen presentation, and stimulate protective CD8⁺ T-cell responses [36]. In addition, senescence can increase tumor-cell responsiveness to environmental IFNγ and strengthen immune-mediated clearance without abolishing the senescence program itself [35]. In vivo liver cancer models further show that senescence surveillance restrains tumor initiation through coordinated immune recruitment, although this benefit depends on prompt and effective clearance of senescent cells [37].

##### DNA damage response (DDR) activates senescence to prevent malignant transformation

The DNA damage response (DDR) is a major upstream trigger of senescence in both precancerous and therapy-exposed cells. Persistent activation of DDR signaling enforces durable proliferative arrest through the ATM/ATR–p53–p21 axis and related checkpoint pathways, thereby limiting the propagation of genomically unstable cells [23, 24]. In cancer cells, ionizing radiation can induce a stable senescence phenotype characterized by SA-β-Gal positivity, lysosomal activation, morphological remodeling, and sustained cell-cycle arrest, illustrating how DDR-driven senescence acts as a tumor-suppressive endpoint after genotoxic stress [27].

##### Aging-associated tissue context

Recent evidence suggests that aging itself may exert tumor-suppressive effects in selected oncogenic settings. In KRAS-driven lung cancer models, aging reduced tumor initiation and growth, weakened the oncogenic advantage conferred by the loss of several tumor suppressors—especially PTEN—and preserved aging-associated transcriptional programs in transformed cells [65].

#### Senescence as a tumor promoter

##### Chronic SASP-driven malignancy

In contrast to its short-term protective role, persistent senescence can become tumor-promoting when senescent cells accumulate and chronically secrete SASP factors. Long-term senescence has been shown to enhance stemness and tumor-initiating capacity after escape from growth arrest [7]. In fibrotic liver disease, the secretome of senescent hepatic stellate cells promotes proliferation, epithelial-mesenchymal transition, and malignant transformation of hepatocyte-lineage cells through oncogenic signaling pathways [21]. Likewise, senescent mesenchymal stem cells remodel the extracellular matrix and drive breast cancer cells toward a more invasive phenotype [18].

##### Metabolic reprogramming-driven malignancy and nutrient competition

Senescent cells remain metabolically active and undergo extensive metabolic rewiring rather than entering a passive dormant state. Therapy-induced senescent cancer cells can even engulf neighboring cells to enhance their own survival, underscoring the existence of an active nutrient-scavenging phenotype [66]. Senescent tumor cells also display exploitable metabolic dependencies, including elevated bioenergetic and proteostatic demands, indicating that metabolic remodeling is a core feature of senescence persistence [31]. Within the broader tumor microenvironment, metabolic competition for glucose and other nutrients can suppress antitumor immune responses [67]; therefore, senescence-associated metabolic rewiring is likely to contribute to functionally hostile niches that support tumor persistence and therapeutic resistance.

##### Immunosenescence

Senescence can also promote cancer through the establishment of an immunosenescent or immunosuppressive microenvironment. Senescent cells may evade immune clearance by upregulating inhibitory ligands such as HLA-E, thereby suppressing NK-cell and CD8⁺ T-cell cytotoxicity [39]. Persistent senescent cells can additionally escape immune recognition through MMP-dependent shedding of NKG2D ligands and paracrine suppression of NKG2D-mediated surveillance [38]. More recently, therapy-induced senescent cancer cells were shown to upregulate PD-L1 through ribophorin 1-dependent glycosylation, thereby promoting immune escape, recurrence, and tumor progression after treatment [49, 68].

##### Epigenetic changes drive malignancy

Senescent cells undergo extensive epigenetic remodeling, including changes in chromatin accessibility, histone modifications, DNA methylation and the formation of senescence-associated heterochromatin domains. Although some of these alterations stabilize cell-cycle arrest, persistent or dysregulated epigenetic remodeling can also sustain SASP programs, enhance cellular plasticity, and promote pro-tumorigenic behavior in neighboring cells or in cells that escape senescence [7, 69, 70]. Therefore, epigenetic evolution within senescent cells is increasingly recognized as a key mechanism linking senescence persistence to malignant progression.

## Tumor cell death

The modalities discussed here were selected based on their established relevance to cancer, growing evidence of interplay with cellular senescence, therapeutic tractability, and recency. Other regulated cell death subtypes recognized by the Nomenclature Committee on Cell Death—including lysosome-dependent cell death, entotic cell death, NETotic cell death, and mitochondrial permeability transition-driven regulated necrosis—as well as more recently emerging programs such as disulfidptosis, are not discussed in depth here, because their interplay with cellular senescence in cancer remains less well characterized; we direct interested readers to recent focused literature [8, 66, 71, 72].

Throughout this review, we use the term regulated cell death, as recommended by the Nomenclature Committee on Cell Death, to denote cell death resulting from failed adaptation to stress that can be modulated pharmacologically or genetically, in contrast to physiological developmental death programs [72].

**Fig. 2:**
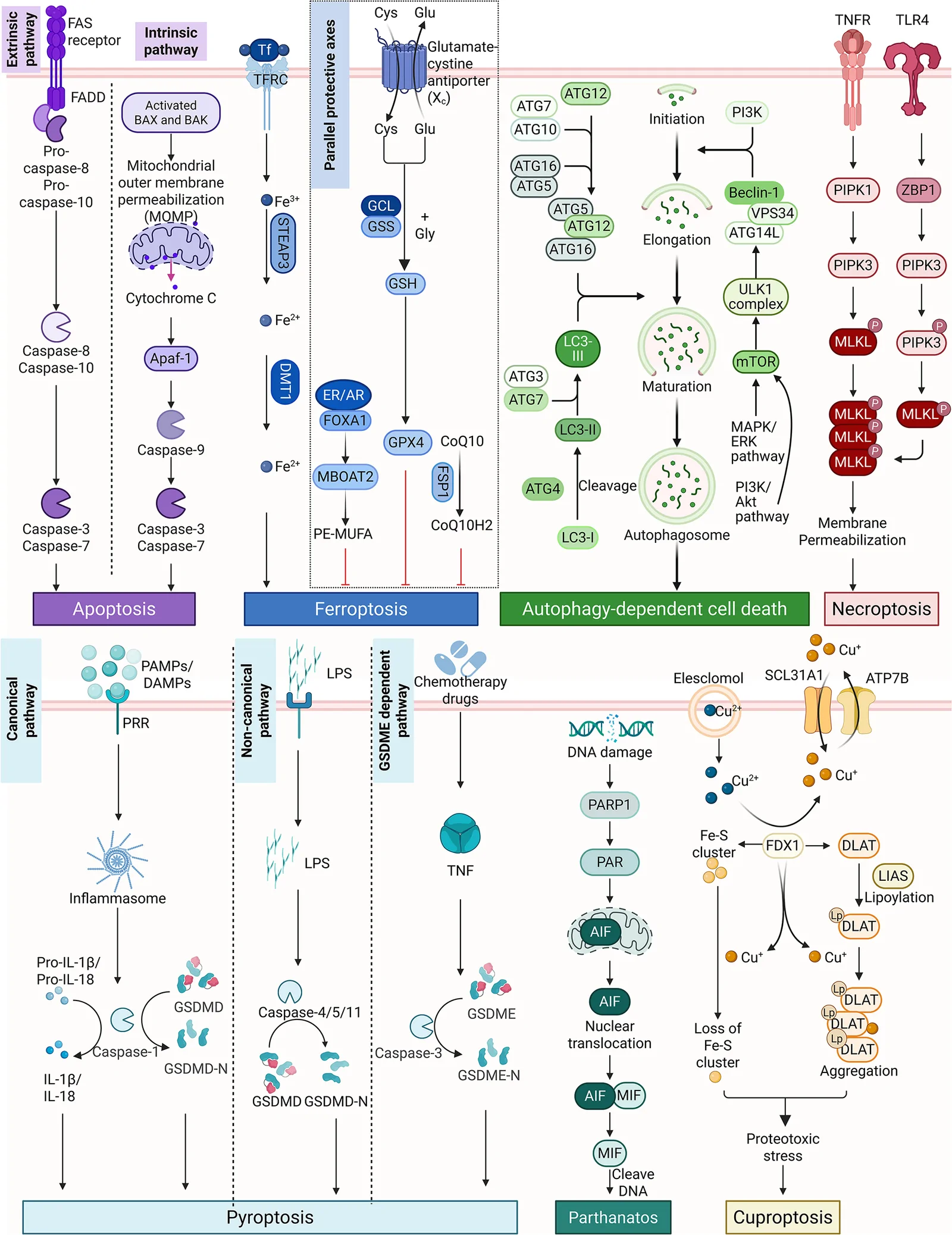
Major forms of regulated cell death in cancer and their core defining machinery. Apoptosis: intrinsic pathway—BAX/BAK → MOMP → cytochrome c → Apaf-1/apoptosome → caspase-9 → caspase-3/7; extrinsic pathway—death receptor → FADD → caspase-8; TLR/NF-κB signaling is not depicted as a canonical execution pathway. Ferroptosis: iron-dependent phospholipid peroxidation is depicted centrally; system xc⁻ mediates cystine uptake and glutamate export, followed by GSH synthesis and GPX4-dependent detoxification of lipid peroxides; FSP1/CoQ10 and MBOAT2 are shown as parallel protective pathways. Autophagy-dependent cell death: the essential machinery (ULK1, Beclin 1, VPS34, ATG5, ATG7, LC3) is shown with an explicit mechanistic-dependency annotation. Necroptosis: TNFR1 → RIPK1 → RIPK3 → MLKL phosphorylation → MLKL oligomerization and membrane translocation → membrane permeabilization; ZBP1 activates RIPK3 through a distinct route; necroptosis critically depends on RIPK3 and MLKL, whereas the requirement for RIPK1 is context-dependent. Pyroptosis: canonical (NLRP3/ASC inflammasome → caspase-1 → GSDMD cleavage → IL-1β/IL-18 maturation), noncanonical (caspase-4/5 in humans, caspase-11 in mice → GSDMD cleavage), and caspase-3/GSDME-dependent branches are shown separately, the latter as a distinct, context-dependent route often downstream of apoptotic signaling. Parthanatos: PARP1 hyperactivation → PAR accumulation → mitochondrial AIF release and nuclear translocation → AIF/MIF-dependent large-scale DNA fragmentation. Cuproptosis: mitochondrial copper accumulation (SLC31A1-mediated uptake) → FDX1-dependent regulation of protein lipoylation → copper binding to lipoylated TCA-cycle proteins such as DLAT → protein aggregation, loss of Fe-S cluster proteins, proteotoxic stress, and cell death

Tumor cell death is no longer viewed as a process limited to classical apoptosis. In cancer biology, multiple regulated cell death programs have now been identified, including apoptosis, ferroptosis, autophagy-dependent cell death, necroptosis, pyroptosis, parthanatos, and cuproptosis. These death modalities differ in their initiating stimuli, signaling circuitry, metabolic requirements, and immunologic consequences, and together they shape therapeutic response, resistance, and tumor–microenvironment interactions. The key signaling pathways involved in these cell death modalities are shown in Fig. 2.

The major regulated cell death modalities relevant to oncology are summarized in Table 3.

**Table 3:** Major regulated cell death modalities in cancer

Death Modality	Key Executioners	Primary Features
Apoptosis	Caspase-3/-9, BCL-2 family, PARP	Programmed, immunologically silent, caspase-dependent
Ferroptosis	GPX4, ACSL4, system Xc⁻, PUFAs	Iron-dependent lipid peroxidation, non-apoptotic
Autophagy	LC3, ATG proteins, lysosomes	Excessive autophagy leading to cell death
Necroptosis	RIPK1/RIPK3/MLKL axis	RIPK3-mediated necrotic death, immunogenic
Pyroptosis	Gasdermin D/E (GSDMD, GSDME), caspase-1/-3	Inflammatory pore-forming death, IL-1β release
Parthanatos	PARP1, AIF, MIF	PARP1-hyperactivation, nuclear AIF translocation
Cuproptosis	FDX1, lipoylated TCA enzymes, copper	Copper binding to lipoylated proteins, proteotoxic stress

### Apoptosis

Apoptosis remains the best-characterized form of regulated tumor cell death and is mediated through the intrinsic mitochondrial pathway and the extrinsic death-receptor pathway. Recent studies show that p53-dependent mitochondrial apoptosis remains a central tumor-suppressive mechanism, whereas altered mitochondrial metabolism can buffer this death response and support survival in cancer cells [73]. In parallel, death-receptor signaling remains therapeutically actionable, as engineered DR5 agonistic extracellular vesicles can directly induce apoptosis in DR5-positive tumor cells and also target immunosuppressive stromal compartments [74]. More recent mechanistic work further indicates that ER-stress-associated CHOP–DR5 signaling can trigger apoptosis independently of p53 phenotype, and that TRAIL sensitivity can be restored through ER-stress-mediated DR5 upregulation or YAP/TAZ-dependent control of TRAIL-R2/DR5 signaling [75–77].

### Ferroptosis

Ferroptosis is an iron-dependent, non-apoptotic form of cell death driven by phospholipid peroxidation [78]. Its core suppressive system is the system Xc⁻–GSH–GPX4 axis, whereas ACSL4 promotes incorporation of polyunsaturated fatty acids into membrane phospholipids and thereby enhances ferroptotic susceptibility. In breast cancer, clinical and translational evidence supports the relevance of ACSL4 and GPX4 as predictive and prognostic markers in the neoadjuvant setting [79]. In ovarian cancer, ACSL1 has been shown to stabilize FSP1 through N-myristylation, thereby increasing resistance to ferroptosis and platinum therapy [80]. Additional recent work demonstrates that suppression of the system Xc⁻/GSH/GPX4 axis can inhibit nasopharyngeal carcinoma metastasis [81], whereas cancer persister cells surviving targeted therapy remain constrained by residual FSP1 activity and can be re-sensitized to ferroptosis by HDAC inhibition [82, 83].

### Autophagy-dependent cell death

Autophagy-dependent cell death (ADCD) refers to a death process in which the autophagic machinery is itself mechanistically required for the lethal outcome [84]. Operationally, this attribution requires that genetic or pharmacological disruption of autophagy—for example, at the level of autophagosome formation (ATG5, ATG7, Beclin 1) or lysosomal degradation—prevents or delays cell death, with appropriate controls excluding predominant apoptotic execution. Recent evidence in hepatocellular carcinoma illustrates this criterion: activation of the DUSP1/AMPK/ULK1/Beclin1 axis drives autophagy-mediated tumor cell death and suppresses malignant phenotypes [85]. By contrast, autophagy that occurs concurrently with cell death, or that is activated as an adaptive, cytoprotective response to lethal stress, is not a death mechanism per se, and its therapeutic targeting should be considered separately. The clinical inhibition of cytoprotective autophagy by hydroxychloroquine-based regimens is therefore discussed in Section “Anticancer strategies targeting cell death modalities” [86–88], rather than as evidence bearing on ADCD.

### Necroptosis

Necroptosis is a lytic and inflammatory form of regulated cell death mediated primarily by the RIPK1–RIPK3–MLKL cascade. In colorectal cancer cells, OSW-1 has been shown to activate RIPK1/RIPK3/MLKL signaling and induce necroptotic death through a RIPK1–p62/SQSTM1 complex [89]. Similarly, acetylshikonin promotes oxidative stress, MLKL phosphorylation, and RIPK1/RIPK3-dependent necroptosis in non-small-cell lung cancer [90]. Beyond cell-autonomous killing, necroptosis-related programs are also linked to tumor immunity: in hepatocellular carcinoma, RIPK1/RIPK3/MLKL-p expression correlates with intratumoral CD3+ and CD8+ T-cell density and clinical outcome [91], while more recent work indicates that ZBP1-mediated stress sensing can enhance tumor immunogenicity under combined anti-HER2 and epigenetic therapy [92, 93].

### Pyroptosis

Pyroptosis is an inflammatory death program executed by gasdermin family proteins. In cancer, GSDME is especially important because caspase-3 can cleave it downstream of chemotherapy-induced apoptosis, thereby converting apoptosis into pyroptosis. In breast cancer, paclitaxel- and anthracycline-based neoadjuvant chemotherapy has been shown to induce GSDME cleavage, DAMP release, macrophage phagocytosis, and enhanced antitumor immunity [94]. In colorectal cancer, mitochondria-targeted photodynamic therapy can trigger ROS/p38/caspase-3/GSDME signaling and sensitize microsatellite-stable tumors to anti-PD-1 blockade; decitabine further augments this effect by reversing GSDME methylation [95, 96].

### Parthanatos

Parthanatos is a caspase-independent death pathway initiated by PARP1 hyperactivation, with subsequent poly(ADP-ribose) accumulation, AIF release, nuclear translocation of AIF/MIF, and large-scale DNA fragmentation. Recent bioinformatic and clinical analyses indicate that parthanatos-related gene programs define biologically distinct breast cancer subtypes and may be associated with poor prognosis, altered immune infiltration, and potential PARP inhibitor resistance [97]. Experimental work in diffuse large B-cell lymphoma further shows that curcumin can induce PARP1-mediated parthanatos with nuclear accumulation of AIF and MIF, and this effect can be enhanced by UVB irradiation [98].

### Cuproptosis

Cuproptosis is a newly defined form of regulated cell death caused by intracellular copper accumulation and direct targeting of lipoylated mitochondrial enzymes. The foundational study demonstrated that copper binds lipoylated TCA-cycle proteins, especially DLAT, causing proteotoxic stress and establishing FDX1 as a key regulator of cuproptotic sensitivity [9]. More recent evidence in lung adenocarcinoma supports this framework and shows that the CEBPA/FDX1 axis can enhance cuproptosis sensitivity, reinforcing the importance of mitochondrial lipoylation control in copper-dependent tumor cell death [99, 100].

## Cross-regulatory networks between tumor cell senescence and death

The decision between senescence and cell death is not binary but is dynamically regulated by a network of molecular hubs that integrate intrinsic and extrinsic signals.

### The battle between senescence and apoptosis: the dual decision of p53

p53 is a central regulator of both senescence and apoptosis, but its functional output is highly context dependent. In a graded DNA-damage model, low-intensity genotoxic stress activated SRC-p38 signaling, sustained survival/senescence programs, and restrained p53 accumulation, whereas high-intensity damage failed to activate SRC and instead permitted robust p53 induction and apoptotic commitment [101]. Genetic dissection of p53 effector pathways further indicates that p21-driven growth arrest/senescence and PUMA-dependent apoptosis are mechanistically separable yet cooperative arms of tumor suppression, because simultaneous disruption of these outputs markedly increased tumor incidence and altered tumor spectrum in vivo [102]. At a finer mechanistic level, the transcriptional output of p53 is influenced by its post-translational modification pattern, with p300/CBP-dependent acetylation at K120/K164 promoting full target-gene activation, whereas SIRT1-mediated deacetylation restrains p53 activity and supports cell survival [103, 104].

Senescent cells are not passive endpoints but actively construct anti-apoptotic circuits that blunt p53-mediated killing. Senescence-associated super-enhancers upregulate MDM2, RNASE4, and ANG to suppress p53-dependent apoptosis and thereby maintain senescent-cell viability [105]. Likewise, in mismatch-repair-deficient colorectal cancer, WRN acts as a brake on the p53/PUMA apoptotic axis [106]. In breast cancer, GD3 synthase was identified as another anti-apoptotic node closely associated with p53 status. Wild-type p53 represses GD3S, whereas gain-of-function mutant p53 increases GD3S expression. In turn, GD3S maintains mitochondrial fitness and counteracts apoptosis induced by wild-type p53 [73].

Recent work also connects p53 status to the inflammatory quality of senescence. p53 activation was shown to enhance DNA repair, suppress γH2AX-positive cytoplasmic chromatin fragment formation, and attenuate downstream inflammatory signaling in senescent cells [107]. Taken together, these findings support a dynamic model in which p21-associated arrest and senescence compete with the apoptotic arm of p53.

### Mitochondria as the core integrating hub

Positioned downstream of, and in parallel with, the DDR decision hub (Section “Hallmarks of cellular senescence”), mitochondria act as a central integrating hub at which metabolic, oxidative, inflammatory, and apoptotic signals converge to determine whether stressed tumor cells maintain a senescent state or progress toward death. Time-resolved proteomic analysis showed that senescence is accompanied by extensive remodeling of the mitochondrial proteome, with accumulation of mitochondria displaying reduced oxidative phosphorylation per organelle but an overall rewired metabolic output, indicating that mitochondrial adaptation is a core feature of the senescent state [108].

Rather than reflecting a single fixed morphological pattern, senescence is associated with context-dependent disruption of mitochondrial dynamics and quality control. In a recent study of lung cellular senescence, senescent cells displayed increased mitochondrial number, reduced mitochondrial size and activity, elevated DRP1 signaling, and decreased MFN2/OPA1 expression, whereas restoration of MFN2 or OPA1 attenuated oxidative stress, SASP factor expression, mitochondrial damage, and senescence markers [109]. These findings support that the balance between fusion, fission, and mitochondrial fitness is a key determinant of whether mitochondria support persistence of senescence or promote collapse of cellular viability.

Mitochondria also function as active signaling platforms that amplify the inflammatory phenotype of senescence. A landmark study showed that senescent cells undergo sublethal mitochondrial apoptotic stress in the form of minority mitochondrial outer membrane permeabilization (miMOMP), which permits BAX/BAK-dependent release of mitochondrial DNA into the cytosol and activates the cGAS-STING pathway to drive the SASP [110]. More recently, a mitochondria-regulated p53 circuit was shown to control cytoplasmic chromatin fragment formation, DNA repair capacity, and inflammatory output in senescent cells, further linking mitochondrial dysfunction to persistent senescence-associated inflammation [107].

Mitophagy is a major protective mechanism that restrains the buildup of damaged mitochondria during senescence. Suppressed basal mitophagy was recently shown to be sufficient to induce senescence phenotypes, whereas pharmacologic reactivation of the PINK1–PRKN–SQSTM1 pathway alleviated aging-associated cellular features [111]. Consistent with the idea that senescent cells remain under chronic mitochondrial stress yet avoid immediate death. Transient Cyclophilin D-dependent opening of the mitochondrial permeability transition pore supports their survival, while its inhibition causes mitochondrial Ca²⁺ overload and senolytic cell death [112].

Mitochondrial metabolism also shapes the chromatin and transcriptional landscape of senescence. Recent work demonstrated that DNA damage signaling can be transmitted to mitochondria to stimulate mitochondrial fatty acid oxidation, which in turn promotes senescence establishment [113]. In parallel, citrate metabolism through ACLY generates acetyl-CoA required for remodeling pro-inflammatory enhancers and recruiting BRD4, thereby sustaining the SASP independently of stable cell-cycle arrest [114].

In summary, mitochondria serve as a central hub in senescence by integrating metabolic, oxidative, inflammatory, and apoptotic signals. Their dynamic remodeling influence whether senescent cells remain viable or progress toward death.

### Transition between senescence and cell death

Four distinct types of relationships between senescence and cell death should be distinguished. First, a stress threshold may determine whether a cell undergoes senescence or death, as when damage intensity and p53 dosage dictate arrest versus apoptosis (Section “The battle between senescence and apoptosis: the dual decision of p53”). Second, a shared upstream factor—such as p53, mitochondrial ROS, or the autophagy machinery—may regulate both states without the cell transitioning between them (Sections “The battle between senescence and apoptosis: the dual decision of p53”, “Mitochondria as the core integrating hub” and “Autophagy as a regulator between senescence and cell death”). Third, paracrine communication allows senescent or dying cells to influence distinct recipient cells, as when SASP factors or pyroptotic mediators shape the fate of neighboring cells (Sections “The consequences of senescence in cancers” and “Mechanistic links between senescence and pyroptosis”). Fourth, a direct transition of the same cell from a verified senescent state into a defined death program occurs only where within-cell conversion has been demonstrated—for example, senolytic-induced apoptosis of verified senescent cells (Section “Senolytics”) and A20–NLRP3–caspase-1–GSDMD-mediated pyroptotic transition of senescent cells under therapeutic stress (Section “Mechanistic links between senescence and pyroptosis”). Throughout this section, the terms “mechanistic links” and “transition” are used accordingly, rather than “interconversion”.

#### Mechanistic links between senescence and apoptosis

**Fig. 3:**
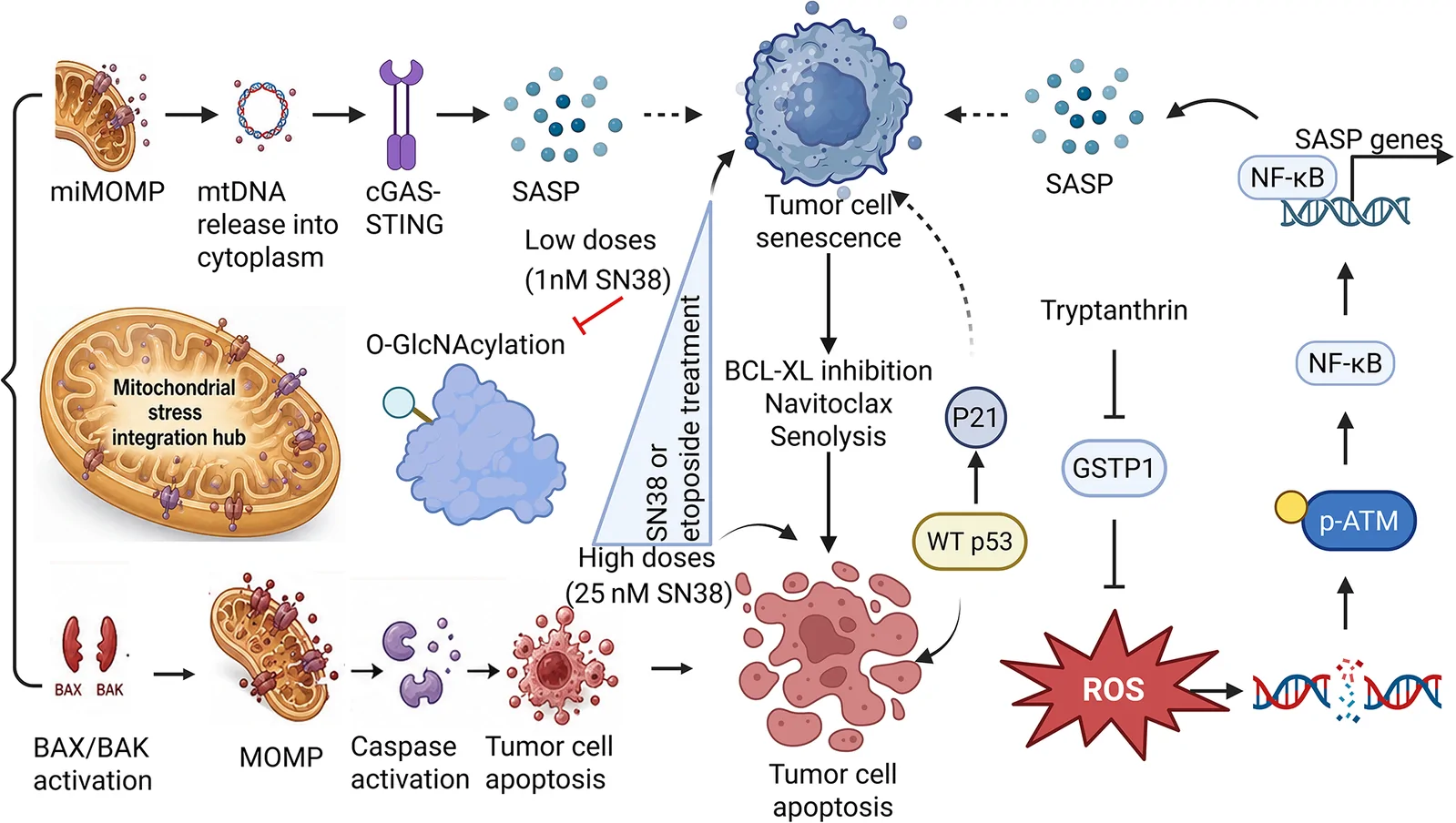
Mechanistic links between cellular senescence and apoptosis. The figure depicts alternative cellular responses to stress rather than true bidirectional interconversion. Extensive BAX/BAK activation drives MOMP and caspase-dependent apoptosis, whereas sublethal mitochondrial stress induces minority MOMP (miMOMP), which reinforces cGAS–STING-dependent SASP signaling in senescent cells rather than directly inducing senescence. The low-dose versus high-dose chemotherapy divergence between senescence and apoptosis is annotated as context-dependent rather than general. The p53 module shows that p53 can promote both cell-cycle arrest and apoptosis, and that p21-mediated arrest is not equivalent to senescence. Senolytic treatment with BCL-xL inhibition or navitoclax induces apoptotic clearance of verified senescent cells. Tryptanthrin regulates the GSTP1/ROS/DDR/NF-κB/SASP axis, exposing a senolytic vulnerability to ABT263

In tumors, senescence and apoptosis should be viewed as competing yet partially interconvertible stress states rather than mutually exclusive endpoints (Fig. 3). Their balance is shaped by damage intensity, mitochondrial apoptotic priming, checkpoint competence, and adaptive survival rewiring [115–117].

In intrinsic apoptosis, extensive BAX/BAK-dependent mitochondrial outer membrane permeabilization commits cells to rapid death, whereas in senescence mitochondrial stress can remain sublethal [118]. A recent high-impact study showed that senescent cells undergo minority MOMP (miMOMP), which releases mtDNA into the cytosol, activates cGAS-STING signaling, and reinforces the SASP. Importantly, blocking BAX/BAK-dependent mitochondrial permeabilization suppresses inflammatory SASP outputs, indicating that incomplete mitochondrial apoptotic stress can actively sustain senescence-associated inflammation rather than simply reflect failed apoptosis [110]. In parallel, tumor studies indicate that metabolic context can determine whether stressed cells consolidate senescence or cross the apoptotic threshold. In liver cancer, tryptanthrin induces GSTP1/ROS/DDR/NF-κB/SASP-dependent senescence while simultaneously exposing a senolytic vulnerability to ABT263-mediated apoptosis [117]. In colon cancer, inhibition of O-GlcNAcylation redirects the response to low-dose chemotherapy from senescence to apoptosis, showing that post-translational metabolic control can gate fate conversion [116].

The clinical relevance of this plasticity has been highlighted in several tumor models. In breast cancer, wild-type p53 does not necessarily improve chemotherapy response; instead, it can favor durable senescence-like arrest over mitotic catastrophe and cell death, allowing residual tumor cells to persist and secrete senescence-associated cytokines with autocrine/paracrine and mitogenic activity [119]. In the same disease context, residual breast tumor cells surviving chemotherapy were shown to activate immune-modulatory programs marked by PD-L1 or CD80, indicating that persistent senescence can be coupled not only to survival but also to immune evasion [120]. Likewise, seminoma cells display a dose-dependent divergence after cisplatin treatment: lower doses promote senescence-like G2/M arrest, whereas higher doses engage Fas/FasL-caspase-8/-3-dependent apoptosis [121]. Importantly, the dose-dependent divergence between senescence and apoptosis is context-dependent rather than general, and p53 can promote both p21-mediated arrest—which is not equivalent to senescence—and apoptotic execution, depending on effector availability and mitochondrial priming [41, 42].

This unstable state is therapeutically exploitable. In TP53-wild-type breast cancer, BH3 mimetics selectively eliminate chemotherapy-induced senescent cells, with BCL-XL or BCL-XL/MCL1 dependence determining senolytic sensitivity [122]. Similarly, ABT-263 clears therapy-induced senescent tumor cells in vitro and in vivo by disrupting BCL-XL-BAX interaction and converting persistent senescence into delayed apoptotic clearance [123]. Accordingly, pro-senescence therapy alone is unlikely to be sufficient unless senescent tumor cells are subsequently removed or rendered susceptible to immune surveillance. Consistent with this view, a recent study demonstrated that chemotherapy-induced senescent cancer cells upregulate PD-L2 to evade immune clearance, whereas PD-L2 blockade restores CD8+ T-cell-driven tumor control and improves therapeutic response [124].

#### Mechanistic links between senescence and ferroptosis

**Fig. 4:**
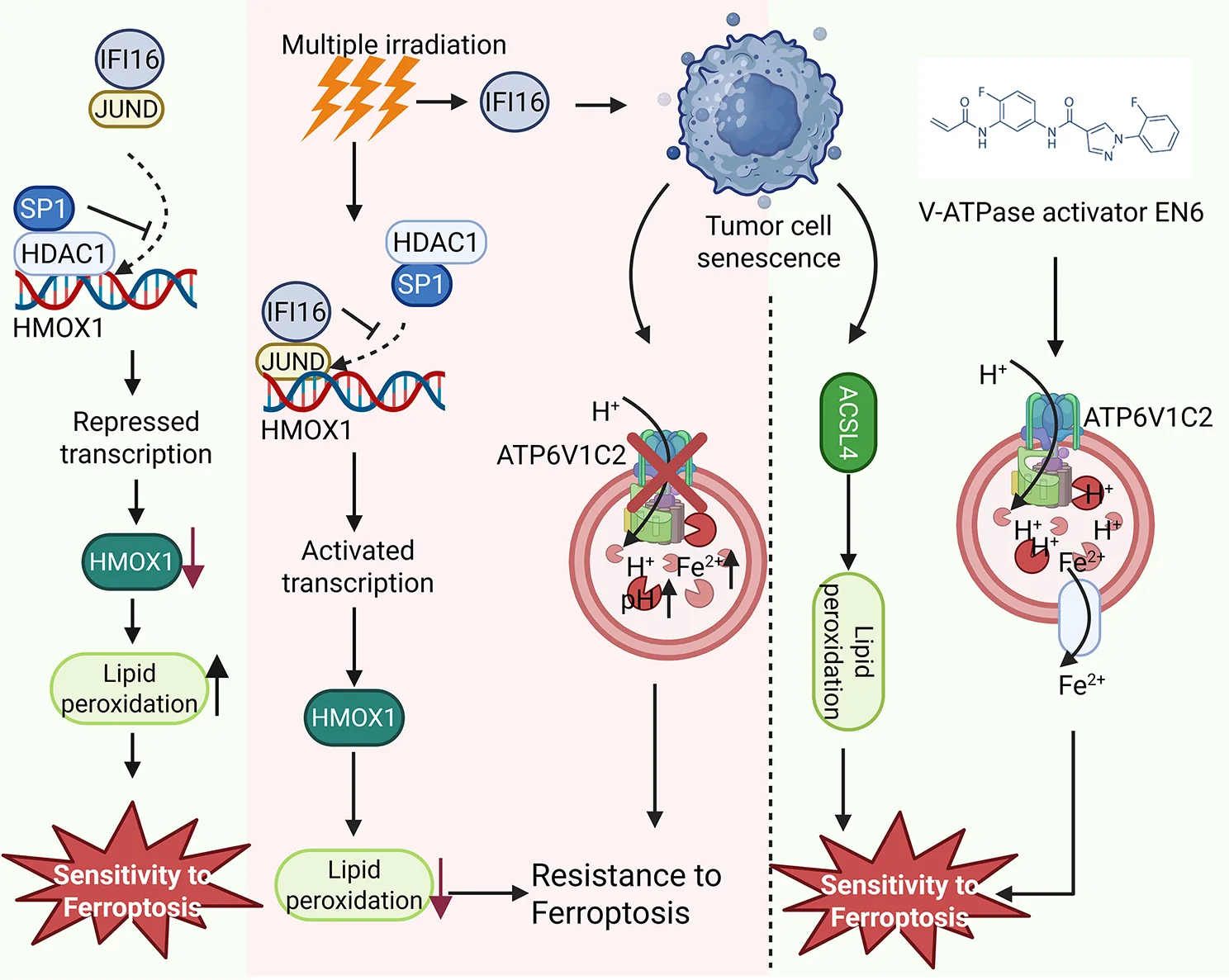
Mechanistic links between cellular senescence and ferroptosis. Activating and inhibitory relationships within the IFI16/HMOX1 and lysosomal iron pathways are shown explicitly. Senescence-associated resistance to ferroptosis (IFI16/HMOX1 signaling; lysosomal iron sequestration) and senescence-induced sensitivity to ferroptosis are distinguished in separate arms. Cotargeting BRD4 and CDK4/6 induces senescence and creates a GPX4-dependent vulnerability that can be exploited to trigger ferroptosis

In tumors, senescence and ferroptosis can convert into each other (Fig. 4). The p53-SLC7A11-GPX4 axis provides a mechanistic basis for this transition. Stabilization of p53 represses SLC7A11 and weakens GPX4-dependent antioxidant defense, thereby lowering the threshold for ferroptotic execution, as shown in triple-negative breast cancer and colorectal cancer models [125, 126]. Ferroptosis competence is further shaped by membrane lipid remodeling. In lung adenocarcinoma, LPCAT3 is transcriptionally driven by YAP/ZEB/EP300 and cooperates with ACSL4 to determine ferroptosis sensitivity, indicating that whether a damaged tumor cell remains senescent or progresses to ferroptosis depends not only on ROS burden but also on whether its phospholipid landscape is permissive for polyunsaturated lipid peroxidation [127].

Cellular senescence can also generate ferroptosis resistance. In glioblastoma, a senescence-associated phenotype induced by repeated irradiation upregulated IFI16, which enhanced JUND/SP1-dependent HMOX1 transcription and thereby reduced Fe²⁺ accumulation, ROS production and lipid peroxidation, ultimately promoting ferroptosis evasion and radioresistance [128]. A related mechanism was reported in senescent cells with lysosomal dysfunction. Senescence-associated lysosomal alkalinization impaired cystine deprivation-induced lipid peroxidation and ferroptosis by trapping ferrous iron in lysosomes [129].

However, this resistant state is not irreversible. Cotargeting CDK4/6 and BRD4 strongly induces senescence but simultaneously creates a GPX4-dependent survival state, so pharmacologic GPX4 inhibition selectively triggers ferroptotic death in these senescent cancer cells [130]. Consistently, a senescence-primed metal-organic framework nanoplatform that combines palbociclib with gallium first induces senescence and then amplifies ferroptotic stress by decreasing GPX4/GSH and increasing ACSL4, Fe²⁺ and lipid peroxidation [131].

Additional regulators further complicate this interconversion. ATM can promote ferroptosis through iron-metabolic control by regulating the MTF1-ferritin/FPN1 axis and labile iron availability [132]. By contrast, STAT3 suppresses ferroptosis in gastric cancer by transcriptionally maintaining GPX4, SLC7A11 and FTH1 [133], whereas PGC1α directly modulates mitochondrial lipid peroxidation and mitochondrial dysfunction during erastin-induced ferroptosis [134]. Taken together, current evidence suggests that the key issue in tumors is not whether senescence and ferroptosis are linked, but which redox, iron-handling, lysosomal and lipid-remodeling switches determine the direction of conversion [135].

#### Mechanistic links between senescence and autophagy

**Fig. 5:**
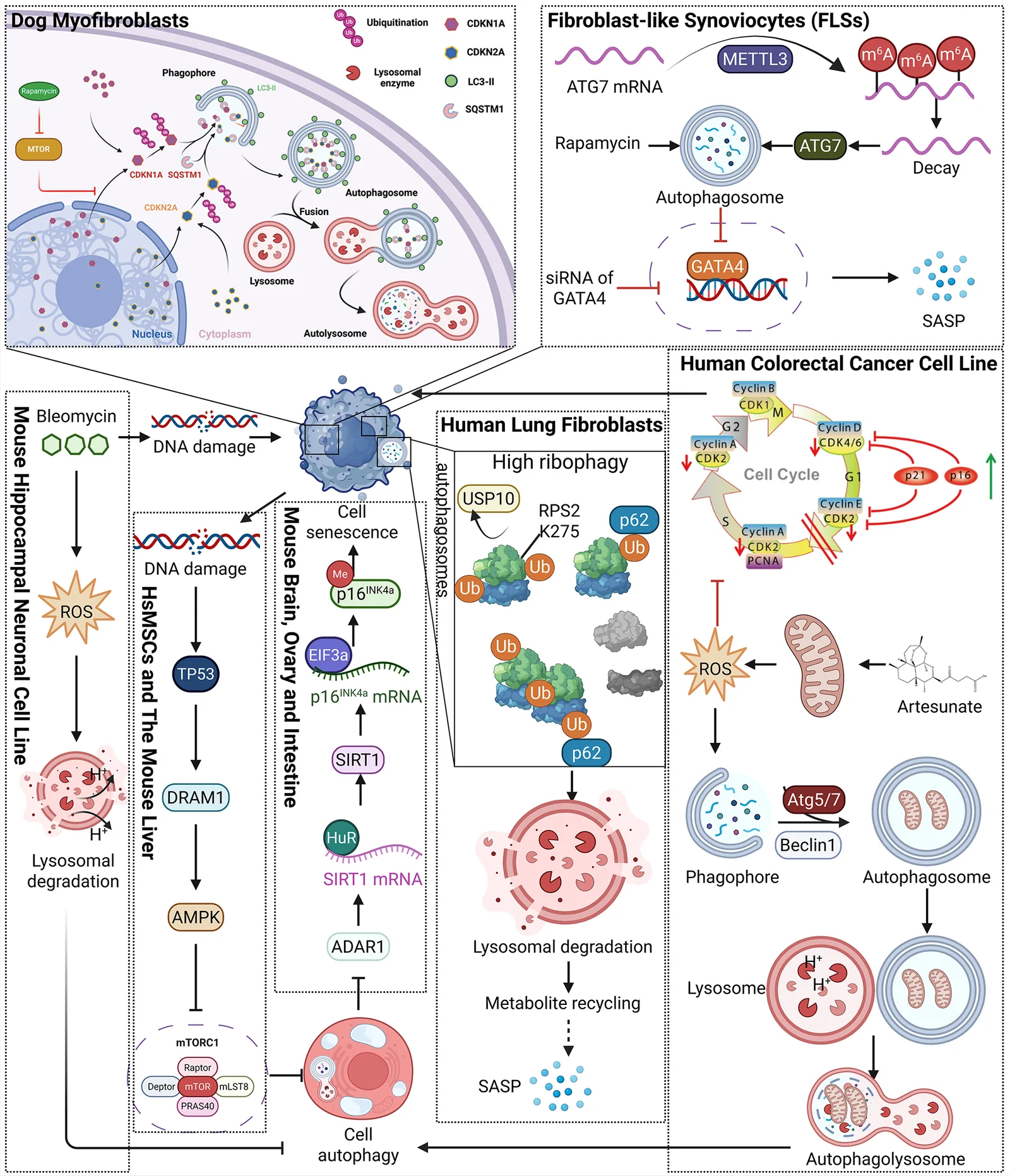
Mechanistic links between cellular senescence and autophagy. Autophagy is not a cell fate that interconverts with senescence; depending on biological context, autophagy can promote, suppress, or maintain senescence. Distinct mechanisms are shown in separate panels labeled with their experimental systems—including p62-mediated degradation of p16 and p21, METTL3/ATG7/GATA4 signaling, ribophagy, lysosomal dysfunction, and artesunate-induced autophagy—and evidence derived from non-cancer models is identified as such

In some settings, autophagy contributes to the establishment of senescence (Fig. 5). During bleomycin exposure, ROS-dependent lysosomal membrane permeabilization and impaired lysosomal degradation occur before overt senescence becomes evident, indicating that lysosomal injury is an upstream event rather than merely a consequence of the senescent state [136]. Autophagic degradation of ADAR1 is likewise sufficient to increase p16INK4a and drive senescence [137].

By contrast, basal autophagy preserves proteostasis and delays premature senescence (Fig. 5). Recent work showed that SQSTM1/p62-mediated selective autophagy can directly sequester and degrade CDKN1A/p21 and CDKN2A/p16, thereby restraining senescence-associated conversion [138]. Chaperone-mediated autophagy (CMA) exerts a similar brake. CMA failure causes PLCG1 accumulation, calcium overload and cellular senescence, whereas UCHL1 stabilizes HSPA8 to sustain CMA and alleviate senescence-linked degeneration [139, 140]. The autophagy-GATA4 axis is another recurring node. In osteoarthritis and intervertebral disc degeneration, restoration of autophagic flux suppresses GATA4 and the SASP through METTL3-ATG7 and KMT2A-METTL3-ATG4A signaling, respectively [141, 142]. Parallel evidence from other tissues supports the same principle. CAV1 promotes renal tubular epithelial senescence by inhibiting CaMKK2/AMPK-driven autophagy, whereas METTL3-dependent destabilization of SIRT1 suppresses autophagy and accelerates senescence in lens epithelial cells [17, 20]. Functional intervention studies further support a protective role for autophagy, as rapamycin reverses radiation-induced pericyte senescence, metformin restores autophagic flux to suppress vascular smooth muscle cell senescence, and cardiac Atg7 deletion is sufficient to induce a senescent phenotype that can later be mitigated by senolysis [143–145].

Senescent cells can also re-engage autophagy as an adaptive program rather than a purely suppressive one. A classic example is the TOR-autophagy spatial coupling compartment (TASCC), in which senescent cells spatially couple autolysosomes with mTOR to sustain high secretory activity and biomass remodeling [146]. More recent work further indicates that aging-rewired metabolic cues can activate DRAM1-dependent pro-senescent autophagy [147].

Autophagy and senescence may therefore be co-induced and jointly shape stress adaptation. In colorectal cancer, artesunate triggers ROS-dependent autophagy together with p16/p21-associated senescence [148]. During oncogene-induced senescence, autophagy also remodels ribosome homeostasis through selective ribophagy, thereby supporting senescence-associated metabolic rewiring and the secretory phenotype [149].

#### Mechanistic links between senescence and necroptosis

In tumors, the relationship between senescence and necroptosis is currently better supported as reciprocal inflammatory crosstalk than as a rigorously established bidirectional cell-fate conversion. Mechanistically, necroptosis is governed by the TNF–RIPK1–RIPK3–MLKL axis and ZBP1–RIPK3 signaling and is tightly constrained by apoptotic checkpoints [150, 151]. Evidence suggests that necroptosis-related machinery can impinge on senescence programs. In hepatocyte-specific hRipk3-KI and hMlkl-KI mice, enforced enhancement of the RIPK3-MLKL module increased inflammatory signaling and was accompanied by significantly elevated markers of cellular senescence in aged liver [152]. Likewise, RIPK1 deficiency caused a RIPK3- and caspase-8-dependent premature senescent phenotype in T cells [19].

Senescent cells establish a SASP-rich inflammatory environment. Such a milieu may facilitate TNF/RIPK-dependent necroptotic responses in neighboring cells when caspase-dependent restraints are weakened [150, 151, 153]. Importantly, in Sod1-knockout mice, senolytic treatment reduced phosphorylated and oligomerized MLKL, whereas pharmacologic inhibition of necroptosis lowered p16, p21 and p53, arguing for a feed-forward circuit between senescence and necroptosis [153]. Consistent with this view, necroptosis inhibition in aged liver also reduced inflammatory cytokines, fibrosis, and senescence-associated features [154]. Therefore, senescence and necroptosis are best viewed as closely apposed stress outcomes embedded in a shared inflammatory signaling landscape.

Mitotic catastrophe deserves particular attention as a potential branching point linking senescence and necroptosis. Mitotic catastrophe arises from aberrant mitosis—typically after mitotic arrest induced by DNA-damaging agents, microtubule poisons, or irradiation—and is increasingly viewed as a tumor-suppressive surveillance mechanism rather than a death program per se. Its outcome is context-dependent: when apoptotic execution is unavailable, cells undergoing mitotic catastrophe can be rerouted toward RIPK1/RIPK3/MLKL-mediated necroptosis; indeed, caspase inhibition or suppression of autophagy promotes RIPK1 phosphorylation and necroptotic death in mitotic-catastrophe cells, identifying apoptosis and autophagy as modulatory pathways that determine the final outcome [155]. Conversely, when checkpoint integrity is preserved—most notably intact p53/p21 signaling—cells can exit the mitotic insult into durable senescence-like arrest instead of progressing to mitotic catastrophe and death, as shown in breast cancer models where wild-type p53 favors senescence-like arrest over mitotic catastrophe [119]. Thus, the fate landscape spanning senescence, mitotic catastrophe, and necroptosis is governed by the same decision logic articulated in Sections “The battle between senescence and apoptosis: the dual decision of p53” and “Mitochondria as the core integrating hub”—DNA damage dosage and repairability, p53 status, and mitochondrial and apoptotic competence—with caspase activity and autophagy acting as modulators that reroute mitotic catastrophe between apoptotic and necroptotic execution.

#### Mechanistic links between senescence and pyroptosis

**Fig. 6:**
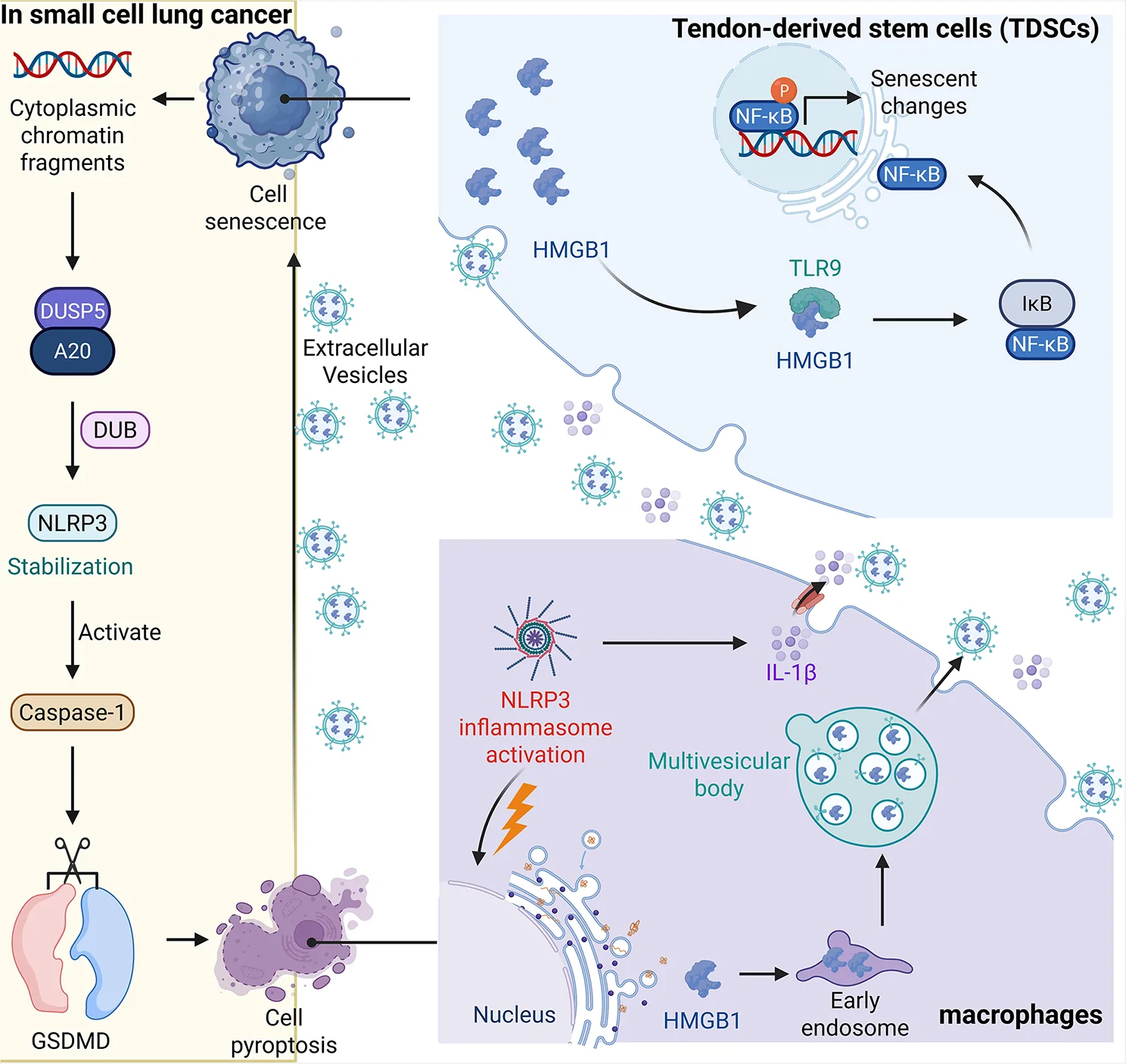
Mechanistic links between senescence and pyroptosis. The transition from senescence to pyroptosis (A20 → NLRP3 → caspase-1 → GSDMD, demonstrated in tumor-cell models under therapeutic stress) is clearly distinguished from pyroptosis-induced paracrine senescence of neighboring cells (IL-1β- and HMGB1-containing extracellular vesicles, demonstrated in non-tumor models). The role of DUSP5 in releasing ERK-dependent inflammatory output is indicated, and NF-κB is highlighted as the common downstream pathway in tendon-derived stem cells responding to HMGB1-containing extracellular vesicles and IL-1β; the HMGB1/TLR9/NF-κB route is presented as context-dependent rather than universal

Senescence and pyroptosis are coupled through both fate transition and paracrine reinforcement (Fig. 6). In small cell lung cancer (SCLC), chemotherapy-induced senescent cells were shown to convert into pyroptotic cells after cisplatin and etoposide treatment. Cytoplasmic chromatin fragments (CCFs) activated the ubiquitin-editing enzyme A20, which stabilized NLRP3 through deubiquitination and drove pyroptosis in a subset of senescent cells. Importantly, pyroptosis-derived inflammatory mediators, especially IL-1, enhanced stem-like properties in the remaining CCF-negative senescent cells and promoted post-treatment recurrence [156].

Additional mechanistic evidence suggests that pyroptosis can also propagate or reinforce senescence in neighboring cells. In a trauma-induced heterotopic ossification model, pyroptotic macrophages induced senescence of tendon-derived stem cells through IL-1β and HMGB1-containing extracellular vesicles [157]. Likewise, GSDME-dependent non-canonical pyroptosis promoted vascular senescence in abdominal aortic aneurysm [158]. In parallel, the NLRP1-GSDMD pathway has been implicated in DNA damage-induced senescence and SASP regulation. NLRP1 acts as a cGAS-dependent DNA-damage sensor during senescence and mediates SASP output through GSDMD [159]. Collectively, these findings support that senescent cells may transition into pyroptotic cells under therapeutic stress, whereas pyroptotic signaling can amplify senescence in surrounding cells through inflammatory paracrine circuits. It should be noted that the senescence-to-pyroptosis transition (A20–NLRP3–caspase-1–GSDMD) has been demonstrated in tumor-cell models, whereas pyroptosis-induced paracrine senescence (IL-1β- and HMGB1-containing extracellular vesicles acting on tendon-derived stem cells, and GSDME-dependent vascular senescence) derives largely from non-tumor models, so these findings should not be presented as a single bidirectional mechanism. In this setting, DUSP5—a negative regulator of ERK signaling—is downregulated, releasing ERK-dependent inflammatory output and facilitating NLRP3 inflammasome activation; in both settings, NF-κB functions as the common downstream pathway.

#### Mechanistic links between senescence and parthanatos

Senescence and parthanatos are functionally linked through shared DNA damage, oxidative stress, and PARP1-centered stress signaling [160, 161]. In colorectal cancer models, AKT inhibition triggered formation of a p53/SIRT6/PARP1 complex, PAR accumulation, AIF nuclear translocation, and bona fide parthanatos [161]. By contrast, in homologous recombination-proficient cancer cells, PARP inhibition induced enlarged morphology, SA-β-gal positivity, and inflammatory SASP-like programs rather than overt cytotoxic collapse, indicating that PARP-linked damage signaling can also stabilize a senescence-like state [160]. A recent study further showed that PARP1 inhibition can convert oxidative injury-induced cell death into senescence [162]. Overall, current evidence supports a close mechanistic coupling between senescence and parthanatos, but not a firmly established bidirectional interconversion in tumors.

#### Mechanistic links between senescence and cuproptosis

Sublethal copper stress may favor senescence-like arrest rather than overt cuproptotic death [9, 163]. Before cuproptosis was formally defined as a distinct copper-dependent death program, subcytotoxic copper sulfate was shown to induce a senescence-like phenotype in human glioblastoma cells, characterized by reduced proliferation, cell enlargement, increased SA-β-gal activity, upregulation of p16 and p21, and downregulation of Bmi-1 [163]. These findings suggest that, when copper stress is insufficient to trigger the lipoylated-protein toxicity program now recognized as cuproptosis, tumor cells may instead enter a stable senescence-like state [9, 163]. Senescence-associated signaling may also create a state less permissive for cuproptosis. In colorectal cancer, CDKN2A, a canonical senescence-associated gene, was identified as a driver of cuproptosis resistance through regulation of glycolysis and copper homeostasis [164]. CDKN2A-high tumor cells displayed enhanced glycolytic activity together with increased copper metabolism and copper ion efflux programs, supporting the view that metabolic rewiring and copper buffering can blunt cuproptotic stress [164]. Thus, low-level copper stress promotes senescence-like arrest, whereas senescence-associated CDKN2A signaling antagonizes cuproptosis.

### Autophagy as a regulator between senescence and cell death

Autophagy operates as a rheostat that determines whether stressed cells remain viable in a senescent state or progress toward death. In particular, selective autophagy helps stabilize senescence by preserving redox homeostasis, limiting proteotoxic stress, and reinforcing senescence-associated inflammatory signaling through regulated degradation of multiple substrates, including KEAP1, translational machinery components, and TNIP1 [165]. At the same time, autophagic flux can also restrain excessive senescence output. Enhanced autophagy promotes SQSTM1/p62-mediated degradation of CDKN1A/p21 and CDKN2A/p16 and reduces SASP intensity, whereas autophagy deficiency leads to their accumulation and aggravates senescent conversion [138]. These findings support that autophagy acts as a tunable regulator of senescence maintenance rather than a uniformly pro- or anti-senescent pathway.

Mitophagy is a major arm of this rheostat. Basal PINK1/Parkin/p62-dependent mitophagy is robust in proliferating cells but becomes markedly suppressed in senescent and naturally aged cells; importantly, inhibition of mitophagy alone is sufficient to trigger the senescence program, whereas reactivation of mitophagy is required for the anti-senescence effects of NAD precursors or rapamycin [111]. In BrafV600E/Pten-deficient melanoma, deletion of the essential autophagy gene ATG7 increases oxidative stress and senescence and thereby restrains melanoma development, showing that autophagy can help tumor cells overcome a senescence barrier [166]. Conversely, when ROS-induced lysosomal membrane permeabilization blocks autophagic degradation, autophagy failure precedes and promotes senescence; in bleomycin models, restoration of autophagy alleviates, whereas worsening autophagy blockade exacerbates, senescence phenotypes [136]. Thus, autophagy and mitophagy set the senescence threshold largely by controlling damaged mitochondria, ROS accumulation, and lysosomal competence [111, 136, 166].

Autophagy can also become a liability once senescent cells become dependent on intracellular recycling for survival. Senescent cells display persistent mTORC1 signaling despite nutrient deprivation, and correcting this abnormal nutrient- and growth-factor-sensing state restores pathway sensitivity and induces senescent-cell death [167]. In parallel, mTORC1 promotes the pro-tumorigenic SASP by enhancing IL1A translation and thereby sustaining NF-κB-dependent IL6 and related inflammatory outputs [168]; earlier work also showed that AKT-driven senescence requires mTORC1-dependent control of p53, underscoring the central position of mTORC1 in linking mitogenic signaling to senescence entry [169]. Combined L-asparaginase and autophagy inhibition selectively eliminated multiple senescent cell types by simultaneously exhausting extracellular and intracellular asparagine supplies [170]. Taken together, moderate autophagy preserves proteostasis, mitochondrial fitness, and senescent viability, whereas defective flux promotes senescence entry [171].

### Epigenetic regulatory networks

Epigenetic mechanisms dynamically shape the transcriptional programs that determine whether stressed tumor cells maintain senescence, escape it, or progress to cell death [172]. DNA methylation plays an important role in this process. Depletion of UHRF1 or DNMT1 caused G1 arrest, SA-β-gal positivity, and SASP expression, indicating that maintenance DNA methylation actively protects tumor cells from entering senescence [70]. In parallel, PRC2-mediated H3K27me3 also suppresses pro-senescent programs in tumors. PRC2 inhibition derepressed CDKN2A/p16, GATA4, and multiple SASP-related genes and induced a senescence-associated phenotype in responsive cancer models, while PRC2-dependent silencing of GATA4 in breast cancer suppressed senescence and promoted tumor progression [173, 174].

HDAC-dependent chromatin remodeling provides another layer of senescence control in tumor cells. In small-cell lung cancer, SAHA induced a senescence-associated secretory phenotype together with cytoplasmic chromatin changes [175]. Consistent with a pro-senescent role of HDAC inhibition in malignant cells, sodium butyrate enhanced resveratrol- or quercetin-induced senescence in human glioma cells, but not in normal astrocytes, showing that HDAC activity can help tumor cells resist senescence-associated arrest [176].

H3K27me3 deposited by PRC2 is another central node connecting chromatin silencing to senescence biology. PRC2 inhibition derepresses high-H3K27me3 targets such as CDKN2A/p16, GATA4 and multiple SASP genes, increases SA-β-gal activity, and promotes macrophage-recruiting inflammatory programs in responsive tumors [173]. GATA4 is epigenetically silenced by the PRC2 complex in breast cancer, and restoration of GATA4 suppresses proliferation while inducing apoptosis and senescence [174]. Outside cancer models, recent work further shows that EZH2-mediated H3K27me3 can directly repress STING, thereby constraining cGAS/STING-driven senescence-associated inflammation [177].

Long non-coding RNAs also play an important role. MALAT1 can recruit EZH2 to silence ABI3BP, thereby suppressing senescence and promoting gallbladder cancer progression [178]. Likewise, HOTAIR enhances H3K27me3 recruitment to the p16 and p21 promoters in NSCLC cells, represses these cell-cycle brakes, and promotes resistance phenotypes, underscoring how lncRNA-PRC2 circuits can oppose stable senescence [179]. MicroRNAs also participate in senescence entry and maintenance. miR-217 directly targets SIRT1 and increases p53-associated tumor-suppressive signaling [180], whereas miR-34a enhances radiation-induced premature senescence in NSCLC cells through c-Myc repression [181]. miR-21 is consistently upregulated in replicative and stress-induced senescence and contributes to growth arrest through CDC25A/NFIB-p21 circuitry [182]. In addition, inflammatory miRNA signaling can propagate senescence between cells. Exosomal miR-155 derived from M1 macrophages induces a senescent phenotype and senescence-associated secretory features through SOCS1-JAK2/STAT3 signaling [183].

Beyond individual regulators such as DNMTs, HDACs, histone marks, and non-coding RNAs, aging-related epigenetic dysregulation may also operate as a systems-level process that progressively destabilizes cell identity and fate control. In KRAS-driven lung adenocarcinoma, aging reprograms tumor evolution through epigenetic activation of the ISR-ATF4 axis, enhancing epithelial and metabolic plasticity, metastatic competence, and glutamine dependence; ATF4 is required for these aging-associated phenotypes and is sufficient to drive dissemination when overexpressed [184].

## Therapeutic strategies: targeting tumor senescence and cell death

### Senescence-targeted therapies

Senescence-targeted therapies have rapidly expanded and now include senolytics, senomorphics, galactose-based prodrugs, proteolysis-targeting chimeras (PROTACs), nanocarrier-based delivery systems, immunotherapy directed against the senescent cell surfaceome, and stem cell-derived extracellular vesicles. Together, these approaches aim either to eliminate senescent tumor cells or to remodel their harmful phenotypes, offering promising opportunities for more precise and effective cancer therapy. Representative therapeutic applications in different cancer types are summarized in Table 4.

**Table 4:** Therapeutic targeting of senescence in different cancer types

Category	Agents	Type of tumor	Targets or drug mechanisms	References
pro-senescence	Docetaxel	Prostate cancer, NSCLC, head and neck cancer, gastric cancer, breast cancer	Tubulin targeting; cytotoxic chemotherapy that can induce therapy-associated senescence	[179]
	Paclitaxel	Breast cancer, ovarian cancer, NSCLC, bladder cancer, Kaposi sarcoma	Tubulin targeting; can induce therapy-associated senescence and senescence-linked resistance programs	[180]
	Nab-paclitaxel	Breast cancer, NSCLC, pancreatic cancer	Albumin-bound taxane; tubulin targeting; class-level pro-senescence activity has been discussed in recent senescence reviews	[181]
	Cabazitaxel	Colorectal cancer	Third-generation taxane; tubulin targeting; can engage p53-linked anti-tumor programs including senescence-associated responses	[182]
	Palbociclib	Breast cancer	CDK4/6 inhibition; induction and maintenance of tumor-cell senescence	[51]
	Ribociclib	Breast cancer	CDK4/6 inhibition; induction and maintenance of tumor-cell senescence	[51]
	Abemaciclib	Breast cancer	CDK4/6 inhibition; induction and maintenance of tumor-cell senescence	[51]
senolytic	Dasatinib+ Quercetin	Melanoma; HCC; other preclinical senescence models	Tyrosine kinase inhibition; mainly studied as part of the D + Q senolytic combination	[183]
	Navitoclax (ABT-263)	NSCLC cell	BCL-2/BCL-XL/BCL-W inhibition; induces apoptosis in senescent tumor cells	[184]
	ABT-737	colon cancer cells	BCL-2/BCL-XL/BCL-W inhibition	[185]
p53-directed senolytic strategy	FOXO4-DRI	NSCLC; broad preclinical senescent-cell models	Disrupts FOXO4-p53 interaction and promotes apoptosis of senescent cells	[186]
	RG7112	Liposarcoma	MDM2 antagonism; p53 pathway reactivation	[187]
senolytic	Ouabain	biliary tract cancer cells	Na+/K+-ATPase inhibition; broad-spectrum senolytic activity against senescent cells	[188]
	Digoxin	metastatic breast cancer	Na+/K+-ATPase inhibition; elimination of senescent/SASP-producing cells	[189]
	Piperlongumine	Thyroid cancer	ROS elevation; PI3K/AKT-related stress signaling; senolytic potential	[190, 191]
senolytic / signaling-targeted	Nintedanib	Breast cancer	JAK2/STAT3 inhibition	[192]
senomorphic	Oridonin	Colorectal cancer	Suppresses SASP while preserving the senescence phenotype; reduces IL-6 and IL-8	[193]
senomorphic / metabolic intervention	Metformin	Head and neck squamous cell carcinoma	Senostatic/senomorphic metabolic intervention; suppresses SASP factors such as IL-6, IL-8, MCP-1, and GRO	[194]
senomorphic / mTOR inhibition	Rapamycin	broader oncologic contexts	mTOR inhibition; suppresses SASP factors including IL-6, IL-8, TNF-alpha, and IL-1alpha	[195]
senomorphic	Ruxolitinib	broad SASP-modulating settings	JAK inhibition; broad SASP suppression including IL-6, IL-10, IL-1beta, MCPs, MMP-12, and VEGF	[196]
	Simvastatin	Breast cancer	HMG-CoA reductase inhibition; suppresses IL-6/CXCL1-dominant SASP	[197]
combination immunotherapy	PD-1/PD-L1 blockade combined with senescence-inducing therapy	Lung adenocarcinoma	Immune checkpoint blockade combined with senescence induction or senolysis; may reverse T-cell exhaustion and enhance anti-tumor immunity	[198]
immune-senescence strategy	IFN-gamma/sPD-1-engineered BMSCs	Lung adenocarcinoma	Engineered cell therapy; reduces Treg abundance and induces tumor senescence via p16 upregulation	[198]

#### Senolytics

Senolytic agents eliminate senescent cells by exploiting the vulnerabilities specific to the senescent state, which are formalized in the concept of senescent cell anti-apoptotic pathways (SCAPs). Upon entering senescence—particularly therapy-induced senescence—cells upregulate multiple anti-apoptotic and pro-survival proteins, including BCL-2 family members, PI3Kδ, ephrins, and p21, which render them resistant to apoptosis and create survival dependencies. Genome-wide transcriptomic profiling first identified these pro-survival networks in senescent cells and demonstrated that silencing individual network nodes selectively kills senescent cells while sparing proliferating and quiescent counterparts; pharmacological targeting of the same nodes yielded the first senolytic combinations, including dasatinib plus quercetin [185–187]. This node-based framework explains senolytic selectivity and its caveat: because SCAP composition varies across cell types and senescence triggers—as illustrated by the differential sensitivity of senescent adipocyte progenitors to dasatinib and of senescent endothelial cells to quercetin—senolytic efficacy is context-dependent, which is directly relevant to their application in tumor cells undergoing TIS [47, 185–187].

Senolytic agents eliminate senescent cells by exploiting the vulnerabilities specific to the senescent state. The BCL-2 family anti-apoptotic proteins (BCL-2, BCL-xL, BCL-w) are consistently upregulated in TIS tumor cells, creating a dependency that BH3 mimetics can exploit [188, 189]. In human glioblastoma, radiation- or temozolomide-induced senescent-like cells were selectively killed by navitoclax and other BCL-xL inhibitors [188]. Consistently, in glioma stem cells, CEP-1347-induced senescence increased BCL-xL expression and created a strong sensitivity to navitoclax at clinically relevant concentrations [189]. In triple-negative breast cancer, talazoparib plus radiation generated a senescent state that was subsequently converted into apoptotic cell death by venetoclax, which also suppressed tumor regrowth in vivo [190]. Compared with these BCL-family-directed strategies, evidence for dasatinib plus quercetin in cancer remains more limited but is growing; in ovarian cancer-bearing mice, this combination reduced therapy-related adipose senescence, improved metabolic dysfunction, and decreased peritoneal and adipose metastasis when combined with carboplatin or olaparib [191]. Fisetin has also shown senolytic potential in glioblastoma, where it enhanced temozolomide-induced killing and reduced the yield of senescent surviving cells [192].

#### Senomorphics

Senescence-modulating agents are not interchangeable, and we use the following terms throughout this section. Senolytic agents selectively eliminate senescent cells (Section “Senolytics”). Senomorphic agents do not primarily kill senescent cells; they selectively attenuate deleterious phenotypes of established senescent cells—especially the SASP—while preserving growth arrest; rapamycin is the prototype, suppressing the secretory phenotype through mTOR-dependent mechanisms [193]. Senostatic agents act on the senescent state itself to stabilize benign arrest and counteract senescence-associated phenotypic escape: in head and neck squamous cell carcinoma, metformin acted as a senostatic agent during CDK4/6 inhibitor treatment, reprogramming the SASP through mTOR and STAT3 inhibition, reducing stemness-associated effects, and improving tumor control [194]. General anti-inflammatory or immunomodulatory drugs suppress inflammatory mediators irrespective of their cellular origin and should not be labeled senomorphic or senostatic unless senescence-selective mechanisms are demonstrated. These categories are operational and may overlap for a given agent, and the classification of individual compounds should therefore be interpreted in the context of the underlying evidence.

Senomorphic agents do not primarily kill senescent cells. Instead, they attenuate the harmful secretory and inflammatory programs of senescent cells while preserving growth arrest. Rapamycin remains a prototype senomorphic, because it suppresses the secretory phenotype of senescent cells through mTOR-dependent mechanisms [193]. In small-cell lung cancer, SAHA induced senescence together with a strong SASP, whereas EZH2 inhibition reduced cytoplasmic chromatin fragment formation, weakened cGAS-STING-dependent SASP production, and enhanced the anti-proliferative effect of SAHA [175]. Rutin is another representative senomorphic agent; it broadly suppressed the SASP by interfering with ATM-HIF1/TRAF6 signaling and improved chemotherapeutic efficacy in preclinical tumor models [195]. In head and neck squamous cell carcinoma, metformin acted as a senostatic agent during CDK4/6 inhibitor treatment, reprogrammed the SASP through inhibition of mTOR and STAT3 signaling, reduced stemness-associated effects, and improved tumor control in xenograft and patient-derived xenograft models [194].

#### Galactose-based prodrugs

Senolytic prodrugs that are selectively activated in senescent cells by their elevated lysosomal β-galactosidase activity represent a promising precision strategy for senescence-targeted therapy [196]. In these systems, a galactose-containing masking group keeps the cytotoxic payload inactive in most normal cells and is cleaved mainly in SA-β-Gal-high senescent cells, thereby improving selectivity and reducing off-target toxicity [197]. This concept was validated by the β-galactosidase-activated prodrug SSK1, which selectively eliminated both mouse and human senescent cells across different senescence-inducing conditions and reduced senescent-cell burden in vivo [196]. Similarly, galactose-modified duocarmycin derivatives preferentially killed senescent cells in a GLB1-dependent manner and reduced β-catenin-positive preneoplastic senescent cells in vivo, supporting the feasibility of β-gal-guided senolysis in neoplastic settings [197].

This platform can also be integrated into a two-step therapeutic design. In one study, the neddylation inhibitor MLN4924 first drove tumor cells into senescence, and the galactose-conjugated prodrug Gal-LIN then preferentially triggered apoptosis in the senescent compartment [198]. These findings suggest that galactose-based prodrugs are not only useful as stand-alone senolytics, but may also function as senescence-responsive killing modules after pro-senescence therapy, allowing more selective clearance of therapy-induced senescent tumor cells [198].

#### Proteolysis-targeting chimeras (PROTACs)

PROTACs use the cellular ubiquitin-proteasome system to induce selective degradation of target proteins and therefore offer potential advantages over conventional occupancy-driven inhibitors, including catalytic and sub-stoichiometric activity [199]. In senescence-targeted oncology, one attractive extension of this concept is to combine targeted protein degradation with senescence-selective activation. Galacto-modified PROTACs, such as Gal-ARV-771 and Gal-MS99, were designed to remain masked until cleavage by SA-β-Gal in senescent cancer cells; these compounds showed higher senolytic indices than their parent PROTACs and, when combined with etoposide, significantly suppressed A549 xenograft growth with limited systemic toxicity [199]. Thus, PROTAC chemistry can be adapted to exploit a core metabolic feature of senescent cells while preserving the mechanistic advantages of targeted degradation [199].

Another important PROTAC direction is the degradation of anti-apoptotic BCL-xL. DT2216, a VHL-recruiting BCL-xL degrader, retained potent antitumor activity while markedly reducing platelet toxicity relative to conventional BCL-xL inhibitors, because platelets express low levels of VHL and are therefore less susceptible to on-target BCL-xL degradation [200]. More recent structure-guided work further generated second-generation dual degraders of BCL-2 and BCL-xL with greater potency than earlier molecules such as DT2216, supporting continued optimization of degrader-based strategies for apoptosis-resistant tumor cells [201]. These findings are particularly relevant to senescence-directed therapy because therapy-induced senescent tumor cells often rely on BCL-2 family survival programs for persistence [200, 201].

For CDK4/6-related strategies, current evidence more strongly supports a sequential model than a direct CDK4/6-PROTAC senolytic model. CDK4/6 inhibition itself is an effective inducer of tumor-cell senescence and can generate a relatively less pro-tumorigenic secretory phenotype than DNA-damaging agents in breast cancer [48]. In liver cancer, combined inhibition of CDK4/6 and XPO1 induced robust senescence, and the resulting senescent cells acquired selective vulnerability to the CRBN-based PROTAC ARV-825 because of increased CRBN dependence [202]. Therefore, CDK4/6-centered therapy may be used to induce senescence first and then create a therapeutic window for selected PROTAC-based clearance strategies [48, 202].

#### Nanocarriers

Nanocarriers can improve the selectivity and safety of senescence-targeted therapy. Many systems use the high SA-β-Gal activity of senescent cells to trigger drug release [203, 204]. Galacto-encapsulated nanoparticles were shown to release cytotoxic drugs preferentially in senescent cells and to improve tumor regression when combined with palbociclib, with lower systemic toxicity [203]. In addition, galactose-functionalized micelles improved the selective delivery of navitoclax to senescent cells and increased its senolytic index [204]. Surface-targeted systems may also be useful. In breast cancer models, 4N1Ks-decorated sphingomyelin nanosystems targeted senescent cells and showed better senolytic activity than the free peptide [205].

#### Immunotherapy based on the senescent cell surfaceome

Senescent cells show major changes in cell-surface protein expression. Surfaceome analysis identified senescence-enriched extracellular proteins that may serve as targets for selective immunotherapy [206]. One validated example is uPAR. uPAR-directed CAR-T cells efficiently eliminated senescent cells and improved treatment outcomes in lung adenocarcinoma models [207]. Senescent tumor cells can also escape immune attack. A recent study showed that therapy-induced senescent cancer cells upregulate PD-L1 through transcriptional and glycosylation mechanisms, which supports immune evasion and tumor recurrence [49]. In addition, senescent cancer cell-derived nanovesicles can function as a personalized cancer vaccine, activate dendritic cells and tumor-specific T cells, and suppress tumor growth and recurrence [208]. These findings support the development of CAR-T cells, checkpoint-based strategies, and vaccine approaches targeting senescence-associated surface features.

#### Extracellular vesicles from stem cells

Stem cell-derived extracellular vesicles (SC-EVs) are emerging as modulators of cellular senescence. MSC-derived small extracellular vesicles reduced senescence markers, suppressed SASP factors, and improved cell function through the miR-146a/Src pathway [209]. iPSC-derived EVs also alleviated senescent phenotypes by reducing ROS and delivering antioxidant factors [210]. Similarly, embryonic stem cell-derived EVs delayed cellular senescence by inhibiting oxidative stress through a fibronectin-integrin-FAK-AKT-GSK3β-Nrf2 pathway [211]. At present, most direct evidence comes from non-tumor models. Therefore, SC-EVs should be considered a promising senescence-modulating strategy, but their role in therapy-induced senescent tumor cells still needs further study.

### Anticancer strategies targeting cell death modalities

Targeting each cell death modality offers distinct therapeutic opportunities [212, 213], as summarized in Table 5.

**Table 5:** Targeted strategies for each cell death modality in cancer

Death Type	Representative Drugs/Strategies	Mechanism	Clinical Status
Apoptosis	Venetoclax (BCL-2), Navitoclax (BCL-2/xL), BH3 mimetics	Inhibit anti-apoptotic BCL-2 family; release BAX/BAK to induce MOMP	Approved (CLL, AML); Phase II/III (solid tumors)
Ferroptosis	Erastin, RSL3 (GPX4i), IKE, sulfasalazine (system Xc⁻)	Inhibit system Xc⁻ or GPX4; cause lipid ROS accumulation	Preclinical; IKE in Phase I trials
Cuproptosis	Elesclomol (Cu ionophore), Disulfiram, ATN-341 (Cu chelator)	Deliver/remove copper; lipoylated enzyme proteotoxic stress	Phase II trials (elesclomol)
Necroptosis	Necrostatin-1 (RIPK1i), RIPK3 agonists, SMAC mimetics	Activate/inhibit RIPK1-3-MLKL axis; trigger DAMP release	Preclinical
Pyroptosis	GSDME activators, demethylating agents (restore GSDME), imipridones (ONC206)	Activate gasdermin pore formation; engage caspase-3/GSDME axis	Preclinical; ONC206 in Phase I
Parthanatos	PARP inhibitors (olaparib, niraparib)	Hyperactivate PARP1; nuclear AIF translocation	Approved (ovarian, breast, prostate)
Autophagic Cell Death	mTOR inhibitors (rapamycin, everolimus), chloroquine/hydroxychloroquine (late autophagy block)	Excessive autophagy or blocked autophagic flux; organelle degradation	Approved (rapamycin: transplant; everolimus: RCC, breast)

Therapeutic inhibition of cytoprotective autophagy constitutes a strategy distinct from autophagy-dependent cell death and remains clinically active: a phase I breast cancer trial showed that hydroxychloroquine combined with continuous low-dose palbociclib and letrozole was feasible and biologically rational [86], whereas a randomized phase II trial in platinum-sensitive relapsed ovarian cancer showed that adding hydroxychloroquine to chemotherapy did not significantly improve response or survival [87]. A separate phase I study combining chloroquine/hydroxychloroquine with carboplatin–gemcitabine in advanced solid tumors confirmed that late-stage autophagy inhibition is clinically testable, although myelosuppression limited dose intensity [88]. These trials target adaptive, cytoprotective autophagy rather than ADCD, and this distinction should be maintained when interpreting their results.

### Combination therapeutic strategies

Combination therapy is becoming a key strategy in senescence-based cancer treatment. A common design is a two-step approach. First, chemotherapy, targeted therapy, or radiation induces therapy-induced senescence (TIS) in tumor cells. Second, senolytic or senostatic treatment is used to remove senescent cells or suppress their harmful secretory phenotype. This strategy aims to keep the short-term anti-tumor effect of senescence while reducing relapse and SASP-driven progression [189, 190].

This concept has been validated in several tumor models. In triple-negative breast cancer, talazoparib plus radiation induced TIS, and venetoclax given after senescence induction markedly increased apoptosis and delayed tumor recovery in vivo [190]. In glioma stem cells, CEP-1347 induced senescence together with BCL-xL upregulation, and navitoclax efficiently eliminated the senescent cell population [189].

Combination with immunotherapy is also promising. Therapy-induced senescent cancer cells can upregulate PD-L1 through transcriptional and glycosylation mechanisms, which promotes immune escape [49]. In irradiated tumors, anti-PD-1 treatment or ribophorin-1 targeting reduced senescent cancer cells and suppressed recurrence, supporting the use of checkpoint blockade after senescence-inducing therapy [49].

Other combinations aim to control SASP while maintaining growth arrest. In small-cell lung cancer, SAHA induced senescence and a strong SASP, whereas EZH2 inhibition reduced SASP production and enhanced the anti-proliferative effect of SAHA [175]. In head and neck squamous cell carcinoma, metformin acted as a senostatic partner for CDK4/6 inhibition by suppressing mTOR/STAT3-dependent SASP signaling and stemness-associated effects [194].

Epigenetic and apoptosis-based combinations are also important. In AML, 5-azacitidine induced NOXA and primed leukemia cells for venetoclax-mediated apoptosis [214]. This mechanistic synergy is consistent with the clinical benefit of azacitidine plus venetoclax in previously untreated AML [215]. In addition, recent work shows that CBX2/EZH2-driven H3K27me3 suppresses ferroptosis in gastric cancer, providing a rationale for combining EZH2 blockade with ferroptosis-based therapy [216].

### Challenges and limitations of senescence-targeted therapies

(i) Toxicity and off-target effects. Systemic elimination of senescent cells risks interfering with beneficial senescence responses, including wound healing and tumor-suppressive barrier functions (Section “Senescence as a tumor suppressor”). Navitoclax illustrates the dose-limiting on-target toxicity of BCL-xL inhibition—thrombocytopenia—which has motivated platelet-sparing derivatives such as DT2216 (Section “Proteolysis-targeting chimeras (PROTACs)”); senomorphic and senostatic agents (Section “Senomorphics”) may likewise affect physiological senescent cells in normal tissues. (ii) Senescence heterogeneity. Senescent cells differ by trigger, cell type, and duration in their markers, secretory output, and SCAP composition, such that a senolytic effective against one senescence state may spare another; transient, immune-clearing SASP and chronic, tumor-promoting SASP (Section “The consequences of senescence in cancers”) further imply that the therapeutic window is time-dependent. (iii) Tumor heterogeneity. Cancer-type-specific and context-dependent factors—including p53 status, which can favor durable senescence-like arrest over cell death (Section “The battle between senescence and apoptosis: the dual decision of p53”)—determine whether senescence induction results in tumor control or in a reservoir of surviving cells. Reliable biomarkers to identify responsive patients and optimal dosing schedules are still lacking (Section “Combination therapeutic strategies”).

It should be emphasized that, apart from autophagy-inhibitor trials (hydroxychloroquine-based regimens [86–88]) and the azacitidine plus venetoclax combination in AML [214, 215], most senescence- and death-targeting strategies discussed above remain at the preclinical stage in oncology. Human data on senolytic agents—including dasatinib plus quercetin, fisetin, and UBX-class compounds—derive mainly from non-oncology indications [191, 192] such as osteoarthritis, idiopathic pulmonary fibrosis, diabetic kidney disease, and physiological aging, or from early-phase oncology studies of their parent scaffolds rather than from senescence-indication trials. For navitoclax, dose-limiting thrombocytopenia arising from BCL-xL inhibition in early oncology studies has motivated the development of platelet-sparing derivatives such as DT2216 (Section “Proteolysis-targeting chimeras (PROTACs)”). Careful distinction between clinical evidence and preclinical evidence is therefore maintained throughout this section, and translation of the senolytic concept into oncology trials will require tumor-type-specific senescence biomarkers, safety data in cancer populations, and well-designed combination regimens.

## Conclusion

Cellular senescence and regulated cell death are not isolated events in tumor biology. Instead, they are closely connected stress responses that together shape tumor cell fate, therapeutic sensitivity, and disease progression. Senescence can act as an important tumor-suppressive barrier by inducing stable growth arrest and supporting immune clearance. However, when senescent cells persist, they may also promote chronic inflammation, immune evasion, tumor recurrence, and therapy resistance through SASP and other adaptive programs. Therefore, senescence should not be viewed simply as a beneficial or harmful state, but as a context-dependent process in cancer.

At the mechanistic level, the balance between senescence and death is controlled by several key hubs, especially p53, mitochondria, autophagy, and epigenetic regulation. These systems do not work independently. Rather, they form an interconnected network with the DDR as a central node that integrates DNA damage, metabolic stress, redox changes, inflammatory signals, and microenvironmental cues. This helps explain why tumor cells exposed to similar stress may undergo different outcomes, such as stable senescence, apoptosis, ferroptosis, pyroptosis, or other forms of regulated death. A better understanding of these cross-regulatory networks is essential for explaining tumor heterogeneity and treatment response.

From a therapeutic perspective, one important lesson is that senescence induction alone may not be enough. In many settings, therapy-induced senescence should be followed by selective clearance of senescent cells or by suppression of harmful SASP signaling. This supports the development of two-step strategies that combine senescence-inducing treatment with senolytics, senomorphics, immunotherapy, or complementary cell-death activation. Such approaches may help preserve the short-term antitumor benefit of senescence while reducing long-term risks such as relapse and microenvironment remodeling.

At the same time, major challenges remain. Senescent tumor cells are highly heterogeneous, and reliable biomarkers are still needed to identify which patients are most likely to benefit from senescence-based therapy. The optimal timing of senolytic intervention, the risk of off-target toxicity, and the influence of the tumor microenvironment also need further study. In addition, different forms of cell death may have distinct immune and metabolic consequences, which should be considered when designing combination therapy.

Future research should move beyond studying senescence or cell death in isolation, and we identify the following specific and testable gaps. (i) Biomarker-guided senolysis: whether senescence marker signatures measurable at single-cell resolution—including surface markers such as uPAR—can prospectively identify patients who benefit from senolytic add-on strategies should be tested in biomarker-stratified trials. (ii) Fate-commitment timing: whether the interval between therapy-induced senescence establishment and immune clearance can be therapeutically widened requires pharmacodynamic studies of senescent-cell kinetics after TIS induction, to define the optimal timing of senolytic intervention. (iii) Mechanistic dependency: for each reported senescence–death link, whether the transition is causally required—for example, whether genetic disruption of ATG5 or ATG7 rescues cells from autophagy-dependent cell death—should be established in isogenic systems. (iv) Spatial validation: whether the paracrine senescence–death signaling proposed here operates within intact tumors should be examined by spatial transcriptomics in paired pre- and post-treatment specimens. Addressing these questions will determine whether the dynamic transition between senescence and death can be exploited as a therapeutic principle rather than a descriptive framework.

## Data Availability

No datasets were generated or analysed during the current study.
